# Identifying potential uses of crowdsourcing in global health, conflict, and humanitarian settings: an adapted CHNRI (Child Health and Nutrition Initiative) exercise

**DOI:** 10.7189/jogh.08.020704

**Published:** 2018-12

**Authors:** Kerri Wazny, Kit Yee Chan, Crowdsourcing CHNRI Collaborators

**Affiliations:** Centre for Global Health Research, Usher Institute of Population Health Sciences and Informatics, University of Edinburgh, Edinburgh, UK

## Abstract

**Background:**

Crowdsourcing, outsourcing problems and tasks to a crowd, has grown exponentially since the term was coined a decade ago. Being a rapid and inexpensive approach, it is particularly amenable to addressing problems in global health, conflict and humanitarian settings, but its potential has not been systematically assessed. We employed the Child Health and Nutrition Research Initiative’s (CHNRI) method to generate a ranked list of potential uses of crowdsourcing in global health and conflict.

**Process:**

94 experts in global health and crowdsourcing submitted their ideas, and 239 ideas were scored. Each expert scored ideas against three of seven criteria, which were tailored specifically for the exercise. A relative ranking was calculated, along with an Average Expert Agreement (AEA).

**Findings:**

On a scale from 0-100, the scores assigned to proposed ideas ranged from 80.39 to 42.01. Most ideas were related to problem solving (n = 112) or data generation (n = 91). Using health care workers to share information about disease outbreaks to ensure global response had the highest score and agreement. Within the top 15, four additional ideas related to containing communicable diseases, two ideas related to using crowdsourcing for vital registration and two to improve maternal and child health. The top conflict ideas related to epidemic responses and various aspects of disease spread. Wisdom of the crowds and machine learning scored low despite being promising in literature.

**Interpretations:**

Experts were invited to generate ideas during the Ebola crisis and to score during reports of Zika, which may have affected the scoring. However, crowdsourcing’s rapid, inexpensive characteristics make it suitable for addressing epidemics. Given that many ideas reflected Sustainable Development Goals (SDGs), crowdsourcing may be an innovative solution to achieving some of the SDGs.

In “The Wisdom of the Crowds,” Surowiecki demonstrated that collective wisdom has the potential to be a very effective and efficient method for solving problems. He cites a historic example by Galton [1] where a crowd of 787 people guess the weight of an ox almost perfectly, and better than any individual expert. The term ‘crowdsourcing’ was coined in 2006 as a method to outsource problems and tasks rapidly and cheaply to a crowd [2] and the field has grown exponentially in the last decade [3,4]. Crowdsourcing has already been used in health, both in research and in practice, and has mainly been used for diagnostics, surveillance, and new discoveries [5]. For example, crowdsourcing has been used to employ laypersons in identifying malaria pathogens through an online gaming system, to administer an epidemiological study in urban India through Amazon Mechanical Turk, and to study clinical populations in psychology [6-8]. Crowdsourcing has shown, in some cases, to improve the accuracy of traditional data collection methods [4,9].

As traditional epidemiological data reporting and warning systems generally have a one- to two-week lag time, sometimes extending to months, real-time reporting is invaluable, especially for outbreaks of severe infectious diseases, such as SARS, Ebola, measles and influenza [10,11]. Crowdsourcing can reduce the lag from hours to minutes and can drastically aid in having a rapid response to outbreaks, preventing further spread [10,11]. Because crowdsourcing is low-cost, it could be particularly useful in global health where financial, human and health systems resources are particularly scarce [6,12]. Common challenges faced in global health, such as poor health systems and resource scarcity, are often exasperated in humanitarian disasters and conflict settings.

Finally, crowdsourcing could aid organisations in targeting interventions accurately. As publications are often delayed by an average of two years [13], funding agencies and donors who are basing funding decisions on published data may be targeting interventions based on outdated data. Crowdsourcing, therefore, may represent a rapid, low-cost avenue for agencies to check-in to ensure the needs of the community are being properly targeted with current data.

While crowdsourcing may offer a potential solution to global health’s challenges in the modes stated above, its potential uses have not been systematically and transparently assessed. Thus, we used the Child Health and Nutrition Research Initiative’s (CHNRI) method to have experts generate and systematically rank potential uses of crowdsourcing against a set of criteria, tailored to crowdsourcing [14].

## PROCESS

The CHNRI method was used to marry opinions of both experts in global health and crowdsourcing to generate and systematically rank potential uses of crowdsourcing in global health. The CHNRI method uses the principle of collective opinion, or wisdom of the crowds, to score ideas against pre-selected criteria which in turn enables donors, policy makers, and programmers to view the strengths, weaknesses and relative ranking of each idea. Over 50 CHNRI exercises have been conducted, the majority of which have been in the areas of maternal and child health, including research priority setting exercises embedded in high-profile series intended to inform policy, such as the Lancet Newborn Series and Lancet Diarrhoea and Pneumonia Series and others exploring universal health coverage, early child development, and quality of care for maternal and child health [15-20].

Experts in both global health and crowdsourcing were invited by email to participate through a combination of snowballing, literature searches and participation in previous CHNRI exercises. Those who did not respond to our first invitation were not followed-up. Those who agreed to participate were provided ‘seed questions’ and explanations of the context of crowdsourcing and global health. 25% of participants were from Asia and 13% from Africa; 34% of participants resided in LMICs. The participants represented a range of viewpoints, including academic, research, programmatic, non-governmental, and donor organisations. A list of participants who scored ideas can be found in Table S1 in **Online Supplementary Document(Online Supplementary Document)**.

94 participants submitted 361 suggestions for potential ideas of applying crowdsourcing in global health. 239 ideas remained once duplicates were removed and similar ideas combined. Ideas were then scored against 7 criteria, developed specifically for this exercise: (i) technological possibility and cost/time of development; (ii) feasibility in a low- or middle-income country; (iii) issues surrounding use; (iv) scale; (v) impact; (vi) equity; and, (vii) cost, time and innovation. The criteria were meant to address issues of technology development and use in LMICs, equity, impact and innovation over current or traditional interventions or data monitoring activities, and respect for local practices and cultural beliefs. Each criterion has three sub-questions and can be found in **Box 1**.

Box 1 **Description of seven CHNRI criteria and sub-questions**

**Criterion 1 – Technological Possibility and Cost/Time of Development**

• Is it possible to create a tool that could support this idea given the current state of technology?

• Would this tool be relatively easy to develop?

• Would this tool be relatively inexpensive to develop?

**Criterion 2 – Feasibility in a Low- or Middle-Income Country**

• Could this tool be used without stable Internet, cell connection or power connection?

• Could this tool be used without requiring expensive technology for the end-user?

• Is there likely to be technology in place to handle the incoming data?

**Criterion 3 – Issues Surrounding Use**

• Could this tool be applied without any ethical concerns, including considerations of respectfulness of local practices and cultural beliefs?

• Is there likely to be enough incentive/motivation for complete and accurate data submission by the user?

• Is it likely that this tool will be easy to use for the end-user?

**Criterion 4 – Scale**

• Is the scale of the problem being tackled by this tool something that has not been amenable to other solutions in the past?

• Would you say that it would be reasonably straightforward to scale-up any population health benefits that this idea may offer?

• Is there potential for this tool to be scalable to large populations?

**Criterion 5 – Impact**

• Will this tool contribute to sustainable change for better?

• Is it clear who would be helped by this tool and how?

• Will this tool have a marked impact on improved health and well-being in the population?

**Criterion 6 – Equity**

• Is this tool likely to improve equity among access to care in the population?

• Is this tool likely to improve equity among quality of care received in the population?

• Is this tool likely to improve equity among distribution of disease burden in the population?

**Criterion 7 – Cost, Time and Innovation**

• Is it likely that this tool could be implemented within a reasonable among of time (eg, less than 1 year) in the population to achieve impact?

• Will implementing this idea save money compared to alternative existing approaches?

• Would this tool offer a genuine and innovative improvement in addressing the problem?

Each expert was assigned to score 3 of 7 criteria against all ideas in order to minimize scorer fatigue. Criteria were assigned in accordance with each expert’s background. In all, 53 experts completed scoring. **Table 1** displays the number of scorers assigned to each criterion, the number of scorers who scored each criterion and the average number of ideas scored within each criterion (as some scorers left blanks). Relative ranking of ideas was calculated using the average across criteria; within-criterion scores and average expert agreement (AEA) were also calculated. Finally, ideas were categorised into themes. The Box S1 **Online Supplementary Document(Online Supplementary Document)** contains further information on the calculations.

**Table 1:** Distribution of scorers across criteria, assigned and scored

Criterion	No. assigned	No. completed	Average ideas scored*
1 – Technological Possibility and Cost/Time of Development	36	18	13.17
2 – Feasibility in a Low- or Middle- Income Country Context	42	23	15.74
3 – Issues Surrounding Use	41	24	16.20
4 – Scale	42	28	17.75
5 – Impact	41	26	17.67
6 – Equity	41	19	14.61
7 – Cost, Time, and Innovation	39	21	15.59

*Average ideas scored differs from number completed as some scorers left blanks where they felt that ideas were not amenable to the criteria.

## OUTCOMES

239 ideas for potential uses of crowdsourcing were systematically scored against 7 comprehensive criteria by 53 experts. Scores ranged from 80.39 to 42.01 and AEA ranged from 0.73 to 0.48. Scores within each criterion ranged as follows: (i) technical possibility and cost/time of development, 0.98 to 0.45; (ii) feasibility, 0.91 to 0.32; (iii) issues surrounding use, 0.98 to 0.51; (iv) scale, 0.79 to 0.26; (v) impact, 0.86 to 0.29; (vi) equity, 0.74 to 0.14; (vii) cost, time and innovation, 0.85 to 0.31. The top 15 ideas, their scores within each criterion, overall scores, AEA, and themes can be found in **Table 2****.** The full list of ideas, their categorisations into themes is available in Table S2 in **Online Supplementary Document(Online Supplementary Document)**.

**Table 2:** Top 15 ideas for potential uses of crowdsourcing for global health overall, with scores in each criterion, RPS and AEA

Rank	Crowdsourcing idea	Category^1^	Tech. possibility	Feasibility	Issues of use	Scale	Impact	Equity	Innovation	Overall RPS^2^	Overall AEA_3_
1.	Use large crowds of health workers to rapidly share information about disease outbreak to ensure a more rapid global response (such as in the recent West African Ebola outbreak).	Epi R	0.87	0.77	0.90	0.79	0.86	0.72	0.73	0.80	0.73
2.	Create an online marketplace where health workers in remote villages could take a picture of a skin condition, for example, using their cell phones and post them to an internet accessible site where dermatologists from around the world could volunteer to 'read' them and give an opinion on their diagnosis. These diagnoses could then be returned to the health care worker by SMS.	Diag	0.90	0.69	0.89	0.77	0.62	0.74	0.77	0.77	0.67
3.	Use churches/mosques, village leaders or community health workers to take the lead in promoting developing a vital registration system.	Data Gen	0.88	0.71	0.94	0.67	0.79	0.64	0.74	0.76	0.70
4.	Use SMS for notification of birth and deaths in real-time, especially in hard-to-reach areas and during conflict/natural disaster.	Data Gen	0.87	0.83	0.88	0.78	0.70	0.56	0.70	0.76	0.68
5.	Ask CHWs to report demographic features of their communities, such as the number of householders with a female head and number of community meetings within a month.	Data Gen	0.95	0.68	0.84	0.71	0.73	0.58	0.81	0.76	0.70
6.	Crowdsource pathology images for research studies.	Res	0.88	0.64	0.87	0.68	0.76	0.55	0.85	0.75	0.68
7.	Ask large crowds of health workers to suggest ways to make labour and delivery safer.	Prob S	0.89	0.91	0.90	0.60	0.61	0.49	0.83	0.75	0.66
8.	Harness the knowledge of CHWs, field workers and stakeholders outside the health arena to solve challenges associated with implementation and scaling-up of programs.	Prob S	0.89	0.66	0.98	0.69	0.66	0.60	0.76	0.75	0.69
9.	Use crowdsourcing to map outbreaks (eg, measles), immunisation dark spots, access local transportation for emergencies, identification of medical resources, etc.	Epi R	0.79	0.68	0.88	0.71	0.75	0.69	0.73	0.75	0.60
10.	Use large crowds of health workers to report on every instance when they witness a maternal or child death, the cause of that death and if the death could have been prevented.	Data Gen	0.98	0.87	0.82	0.61	0.69	0.59	0.61	0.74	0.65
11.	Have experts contribute voluntarily to develop freely available online training courses (or get national funding to deliver printed materials) that teach documented, high-impact skills, which are in short supply. Design the courses with the intent to let members of communities in need learn to solve problems in their own communities. These educational materials should likely be broad in scope, including basic management or business practices to ensure that the service can be kept running in a sustainable way.	Edu	0.88	0.67	0.84	0.71	0.72	0.62	0.74	0.74	0.64
12.	Epidemic identification in remote environments through collection and analysis of crowd-sourced detection. Healthcare workers (and possibly ordinary individuals) are asked to report new/unusual diseases using mobile phones (eg, taking photographs) to central server which then compiles and analyzes the data.	Epi R	0.83	0.58	0.85	0.74	0.83	0.67	0.66	0.74	0.64
13.	Ask large crowds of global health workers to report through text messages on hours/d spent on travel and conferences/workshops in a specified period and to suggest how they could have learned/contributed without travel in order to find solutions to reduce the carbon footprint and increase efficiency in global health.	Prob S	0.86	0.79	0.82	0.68	0.64	0.64	0.74	0.74	0.63
14.	Use large crowds to assess community demand. Encourage communities to share by SMS a service they believe needs to be in place to support health and development in their community as an opportunity to explore demand-side push for services at a larger scale.	Prob S	0.94	0.73	0.90	0.68	0.67	0.53	0.69	0.73	0.62
15.	Use large crowds of diagnostic tool makers and other scientists to generate new, accurate and reliable, low cost, non-invasive, scalable methods for measuring nutrition-relevant issues such as gestational age and size at birth, low birth weight, length or height, exclusive breastfeeding, usual dietary intake and other forms of nutritional status (eg, multiple micronutrient deficiencies).	Nov Dis	0.78	0.68	0.92	0.74	0.67	0.66	0.69	0.73	0.66

Epi R – Epidemic Responses, Data Gen – Diagnostics, Res – Research, Prob S– Problem Solving, Edu – Education, RPS – Research Priority Scores, which are an average of scores across criteria by all experts who have scored, AEA – Average Expert Agreement, which are an indication of collective optimism of the crowd, and are the frequency of the mode (most common score divided by the total number of scores)

Themes identified include epidemic response, problem solving, data generation, research, education and novel discoveries. Overwhelmingly, the ideas fell into the themes of problem solving (n = 112) and data generation (n = 91). Ten ideas related to epidemic responses, including the top-ranked idea. Eight ideas related to research and to education, seven to diagnostics, and three to novel discoveries.

Using large crowds of health workers to rapidly share information about a disease outbreak, ensuring a more global response, ranked first. Its score was higher than all others by 3 points, and also had the highest overall agreement (AEA = 0.73). This idea was the only to score in the top 25th percentile across all seven criteria. Four other ideas in the top 15 also related to containing communicable diseases; another related to the theme of epidemic responses, one to research, another to access to health and the final to data generation. The criteria that drove the high scores for these ideas were technological possibility and cost/time of development and issues surrounding use, which were either the first or second highest scores for all five of the ideas. Three of the five ideas had their lowest scores in the equity criterion.

Experts felt that crowdsourcing would be a feasible platform for vital registration. The registration could be developed through involvement of community or religious leaders or CHWs, or over SMS in the case of hard-to-reach areas or during conflict or natural disaster. These ideas fell into the theme of data generation and scored highest in issues surrounding use and technical possibility and cost/time of development, but they scored low on equity.

Ideas involving improving maternal and child health were also identified in the top 15. These fell into the categories of problem solving and data generation. They scored high in technical possibility and cost/time of development and feasibility. Despite ranking 7th overall, one idea had a score of only 0.49 in equity.

One idea involving novel discoveries ranked highly, seeking to find a novel way to measure nutritionally relevant issues. This idea’s high score was driven by the issues surrounding the use criterion, where it scored 0.92, and it had fairly unremarkable scores in other criteria.

A sub-group analysis was conducted on ideas relevant to humanitarian or conflict settings, and the top 15 ideas in these settings are displayed in **Table 3**. As these ideas are taken from the same list of general ideas, many overlap with the overall top 15. Six of the ideas were categorised as epidemic responses, six as data generation, two as education and one as problem-solving. Many of the ideas in this category focused on disease spread. Ideas in this domain, for the most part, ranked highly in issues surrounding use and technological possibility and cost/time of development and scored lowest in equity, feasibility, and impact, respectively. The range of AEA for the top 15 conflict ideas was 0.73 to 0.58.

**Table 3:** Top 15 ideas for potential uses of crowdsourcing for global health within conflict, with scores in each criterion, RPS and AEA

Conflict Rank	Crowdsourcing idea	Category^1^	Tech. poss.	Feasibility	Issues surrounding Use	Scale	Impact	Equity	Innovation	RPS^2^	AEA^3^
1.	Use large crowds of health workers to rapidly share information about disease outbreak to ensure a more rapid global response (such as in the recent West African Ebola outbreak).	Epi R	0.87	0.77	0.90	0.79	0.86	0.72	0.73	0.80	0.73
2.	Use churches/mosques, village leaders or community health workers to take the lead in promoting a vital registration system.	Data Gen	0.88	0.71	0.94	0.67	0.79	0.64	0.74	0.76	0.70
3.	Use SMS for notification of birth and deaths in real-time, *especially in hard-to-reach areas and during conflict/natural disaster.*	Data Gen	0.87	0.83	0.88	0.78	0.70	0.56	0.70	0.76	0.68
4.	Use crowdsourcing to map outbreaks (eg, measles), immunization dark spots, access local transportation for emergencies, identify medical resources, etc.	Epi R	0.79	0.68	0.88	0.71	0.75	0.69	0.73	0.75	0.60
5.	Ask men who has sex with men to anonymously report outbreaks of diseases within their circles, such as sexually transmitted intestinal infections (shigellosis, amebiases, etc.)	Data Gen	0.86	0.57	0.96	0.72	0.65	0.57	0.79	0.73	0.68
6.	Gather data on disease spread – health workers reporting on diagnoses through their work day (tracking spread of disease and trends).	Data Gen	0.87	0.67	0.92	0.66	0.62	0.66	0.68	0.73	0.63
7.	Create a platform that can crowdsource diverse skills (incl. engineers, statisticians, computer scientists, psychologists, marketing professionals, scientists and HCWs) to solve bottlenecks that arise in the health care value chain, ranging from medical supply and logistics for vaccines, essential medicines, demand creation to quality of care.	Prob S	0.83	0.59	0.87	0.71	0.73	0.62	0.72	0.72	0.63
8.	Get feedback from affected people in a crisis regarding the humanitarian response, what were their needs, were they met, etc.	Epi R	0.88	0.70	0.84	0.65	0.54	0.73	0.73	0.72	0.63
9.	Have staff report via SMS the last time they were trained on a specified topic.	Edu	0.82	0.83	0.87	0.67	0.60	0.62	0.65	0.72	0.65
10.	Use large crowds of school teachers and/or community leaders to report on gender-based violence (via SMS) either in the home or in public spaces, as an alternative to surveys.	Data Gen	0.89	0.84	0.88	0.71	0.68	0.48	0.58	0.72	0.65
11.	Could crowd sourcing be used to diffuse conflicts and de-escalate tensions in a trouble spot? This may be a simple (managed) social media exercise but more thought is needed.	Prob S	0.86	0.68	0.81	0.58	0.73	0.63	0.71	0.71	0.62
12.	Use mobile phone feedback to rapidly map disease outbreaks.	Data Gen	0.78	0.72	0.73	0.77	0.76	0.58	0.63	0.71	0.63
13.	Use mobile phone feedback to map disasters by scale, location and to assess needs.	Epi R	0.73	0.74	0.84	0.68	0.75	0.54	0.67	0.71	0.61
14.	Ask large crowds of CHWs to report unusual cases and infectious disease of public health concern for early detection of outbreaks (cholera, malaria, haemorrhagic fever, contact tracing in Ebola epidemic, etc.)	Epi R	0.63	0.72	0.93	0.65	0.68	0.63	0.70	0.70	0.58
15.	Crowdsource health workers to come up with innovative solutions to accelerate eradication of polio in countries like Pakistan, Afghanistan and Northern Nigeria through SMS surveys.	Epi R	0.82	0.77	0.92	0.65	0.58	0.47	0.64	0.69	0.62

Epi R – Epidemic Responses, Data Gen – Diagnostics, Res – Research, Prob S– Problem Solving, Edu – Education, RPS – Research Priority Scores, which are an average of scores across criteria by all experts who have scored, AEA – Average Expert Agreement, which are an indication of collective optimism of the crowd, and are the frequency of the mode (most common score divided by the total number of scores)

The bottom 15 ideas can be found in **Table 4**. The lowest ranked idea (RPS = 0.42, AEA = 0.58) was to ask families where they are seeking care and how much they have spent; this idea was categorised as data generation. It scored the lowest in scale (0.26) and equity (0.28).

**Table 4:** Bottom 15 ideas for potential uses of crowdsourcing for global health, with scores in each criterion, RPS and AEA

Rank	Crowdsourcing idea	Category	Tech. Poss.	Feasibility	Issues Surrounding Use	Scale	Impact	Equity	Innovation	RPS	AEA
225.	Use crowdsourcing to revamp vital registration in areas that do not have VR systems in place and also by cross-checking information for quality assurance in areas that have deficient VR systems. For example, individuals (health workers and members of the community – informed via media campaigns) would be encouraged to report births and deaths by text message and an automated server would take care of gathering necessary information to characterize the event (by using a decision tree that would reply with the relevant follow-up information)and to reconcile duplicated reports of events and also verifying accuracy and veracity by texting other phones in the area (identified via geolocation) to verify the information).	Data Gen	0.64	0.32	0.68	0.59	0.54	0.50	0.35	0.52	0.51
226.	Use crowdsourcing to monitor trends in scale-up and coverage of newly introduced vaccines and micronutrients.	Data Gen	0.45	0.59	0.65	0.59	0.52	0.37	0.40	0.51	0.48
227.	Ask large crowds of local health workers (or whatever cadre of workers provides vaccinations) to report by SMS the children that they vaccinate each day to generate real-time data for vaccine coverage.	Data Gen	0.58	0.58	0.59	0.41	0.52	0.29	0.61	0.51	0.49
228.	Monitor phase IV drug trials through crowdsourcing.	Res	0.58	0.58	0.59	0.41	0.52	0.29	0.61	0.51	0.49
229.	Ask large crowds of health workers to better understand why young children have poor appetites and do not grow well.	Data Gen	0.52	0.41	0.56	0.52	0.42	0.52	0.50	0.51	0.50
230.	Use crowdsourcing to help annotate medical data, find errors, outliers, etc., and use this to train algorithms.	Data Gen	0.50	0.70	0.52	0.54	0.48	0.39	0.39	0.50	0.50
231.	Use large crowds of local health experts to collect through SMS what prevents vulnerable women and children from using basic health services, such as breastfeeding, ORS and immunization. Use this information to develop approaches to increase awareness, acceptance and utilization of these services.	Prob S	0.61	0.65	0.66	0.44	0.40	0.34	0.38	0.50	0.54
232.	Ask the general public to submit short code SMS to report malpractice in the health sector, such as illegal under the counter fees, bribes, health staff absenteeism, inappropriate behavior, etc. (similar to U-report in Uganda).	Data Gen	0.64	0.42	0.71	0.56	0.42	0.14	0.54	0.49	0.57
233.	Create an organization like Avon where women come to the home to do monthly product descriptions (like “kitchen parties”) and ask women in those groups to say what they think would make the biggest difference to their children’s health.	Prob S	0.62	0.48	0.62	0.46	0.36	0.36	0.50	0.49	0.49
234.	Use large crowds of community leaders to report vaccine preventable diseases by SMS.	Data Gen	0.61	0.43	0.57	0.54	0.52	0.23	0.51	0.49	0.53
235.	Ask large crowds of global health experts to provide materials (reagents, equipment, etc.) they no longer use or need but that could be redistributed in places where they are needed.	Prob S	0.65	0.68	0.57	0.40	0.39	0.24	0.43	0.48	0.57
236.	HCWs could text photos of suspected cases of NTDs to specialists, yielding population-level data on incidence. Families can be asked to provide dietary information; this can be used to identify additional foods to be fortified.	Data Gen	0.74	0.49	0.77	0.39	0.29	0.26	0.39	0.47	0.58
237.	Give mothers around the world extra phone top-up credit for submitting good and plausible ideas of how to improve their children’s health.	Prob S	0.64	0.44	0.51	0.45	0.31	0.35	0.48	0.45	0.49
238.	Use crowdsourcing to locate unpublished research and evaluation results and ongoing research (who’s doing what before it’s published).	Res	0.51	0.41	0.59	0.43	0.40	0.30	0.44	0.44	0.55
239.	Ask patients and families where they are seeking care, why and how much they are spending.	Data Gen	0.60	0.41	0.69	0.26	0.38	0.28	0.31	0.42	0.58

Epi R – Epidemic Responses, Data Gen – Diagnostics, Res – Research, Prob S– Problem Solving, Edu – Education, RPS – Research Priority Scores, which are an average of scores across criteria by all experts who have scored, AEA – Average Expert Agreement, which are an indication of collective optimism of the crowd, and are the frequency of the mode (most common score divided by the total number of scores)

The lowest 15 ideas scored low across all criteria. Most notably, an idea based on an already successful crowdsourcing application (U-Report in Uganda) ranked in the bottom 15, at #232, had an RPS of 0.49, and scored lowest in equity (0.14). This score was the lowest score received by any idea for any criterion. The idea asks that people submit an SMS to report poor behaviour in the health sector, such as bribes, counter fees, and absenteeism. A similar idea asking to counter corruption in the health sector more generally scored much higher at #102; this idea scored higher in equity as well (0.51).

## INTERPRETATION

This was the first application of the CHNRI method, which is in itself a crowdsourcing-based exercise, to explore potential uses of crowdsourcing in global health and in conflict settings. This exercise brought together expertise from experts in global health and in crowdsourcing, using their collective opinions to systematically and transparently generate and score ideas against tailored criteria.

The AEA in our exercise was fairly low, ranging from 0.73 to 0.48. This could be a result of combining different expertise, from the global health and crowdsourcing communities, or be reflective of crowdsourcing being a new field, or that other exercises have generated ‘research priorities,’ which there may be more consensus on.

The vast majority of ideas fell into the categories of problem solving or data generation (n = 112 and n = 92, respectively). As crowdsourcing is often used to fill a knowledge gap, complete a task or solve a problem, this is not surprising. However, as other categories included novel discoveries, research and diagnostics, which current crowdsourcing companies, competitions, and pilots are aimed at, it is surprising that there were so few ideas proposed in these areas. Indeed, major drug companies currently use crowdsourcing competitions to produce novel discoveries as a means to cheaply conduct research on new interventions [21]. In previous CHNRI exercises, novel discoveries have often scored low due to short timeframes [15]; however, our exercise did not stipulate any timeframe. Not only did two of three novel discoveries score low, but also only three were suggested. This should be investigated in further exercises.

Even in many of the top-ranked ideas, the equity criterion scored low. Upon further examination, despite our best efforts, the wording of the sub-questions for equity may have not been worded to suit the proposed ideas. For example, while an idea might be considered equitable overall, it may not specifically result in improving equity for quality of care *and* access to care *and* distribution of disease. To have an impact on all three of these characteristics would be excellent, but would require quite a comprehensive intervention.

The top-scored idea, to use crowdsourcing to share information regarding disease outbreaks and epidemics, could be related to the timing of the exercise. The experts were invited to submit their ideas shortly after the Ebola crisis, where the slow response was catastrophic and widely criticised [22,23]. During the scoring phase, early reports of Zika were surfacing. However, crowdsourcing is an ideal method to spread information, especially regarding early disease reporting in hard-to-reach or remote areas. Various crowdsourcing platforms have already been successfully used in disaster and humanitarian response settings, such as Ushahidi, Frontline SMS and Geochat [24]. Ushahidi is an open-source crowdsourcing application originally developed to respond to election violence in Kenya. It collects geotagged reports via SMS, voice, and email. The ideas that ranked highly in the conflict-relevant list (**Table 2**) are, naturally, particularly amenable to being addressed by Ushahidi and similar platforms, which have already been used in similar situations.

Interestingly, although ‘wisdom of the crowd’ type of crowdsourcing was predominant in a recent literature review of crowdsourcing in health [5], especially crowdsourcing in diagnostics, the experts overall did not score these ideas as the most promising uses. Additionally, many of the uses of crowdsourcing identified in the literature review specifically combined crowdsourcing with machine learning, fine tuning algorithms to merge results from humans and computers to create the best predictive result; these appeared to be among the most successful instances of crowdsourcing. Only one idea, #230, suggested to use crowdsourcing to train algorithms, and although it does not specifically mention machine learning, this appears to be implicit. Interestingly, this idea scored quite low in technological possibility and cost/time of development (0.50). This may be indicative to challenges of bringing two different fields together.

Within the conflict/humanitarian related results, #11 asks whether crowd sourcing could be used to de-escalate tensions or diffuse conflicts in a trouble spot. This idea highlights the potential to use social media to mobilise crowds to achieve a goal. Interestingly, since the exercise’s completion, a movement called the “Injustice Boycott” has formed in the United States in response to the United States election, alongside Black Lives Matter and the Dakota Access Pipeline protests. The Boycott is e-mail and social media based, has mobilised over hundreds of thousands of people and produced real results, including pressuring the city of Seattle to divest from Wells Fargo [which supported the Dakota Access Pipeline), pressuring the Governor of New York to raise the mandatory minimum age for children to be tried as adults, to compel the Golden State Warriors to fire an employee known for overt racism and raising half a million USD for Standing Rock [25]. The Injustice Boycott is an example of how social media can be used to mobilise people to influence government and organisations to produce palatable results.

While this exercise was not primarily focused on the Sustainable Development Goals (SDGs), we noticed that many of the ideas generated relate to a wide range of the SDGs. In the top 15, ideas relate predominantly to the SDG 3.3, addressing epidemics and eliminating communicable diseases. Other SDG targets found in the top 15 include SDG 16.9 (providing legal identity for all, including birth registrations), SDGs 3.1 and 3.2 (reducing maternal and child mortality), SDG 4.3 (increasing the number of youth and adults with relevant skills and training for employment), SDG 3.8 (achieving universal health care and increasing assess to quality health care, essential medicines and vaccines) and, finally, SDGs 2.1 and 2.2, which focus on ending hunger and malnutrition (with the latter focusing on vulnerable populations and periods of strategic growth). In the conflict-specific list, using crowdsourcing to address SDG 1.5 (building resilience of the poor and vulnerable, reduce exposure to climate-, economic-, social-, environmental-related shocks and other disasters), SDGs 5.2 and 5.3 (eliminating violence against women and girls, and eliminating harmful practices such as early and forced marriage and female genital mutilation), SDG 11.5 (significantly reduce the number of deaths and people affected and substantially decrease the direct economic losses caused by disasters), SDG 13.1 (strengthen resilience and adaptive capacity to climate-related hazards and natural disasters), SGD 16.1 (significantly reduce all forms of violence everywhere) and SDG 16.2 (end abuse, exploitation, trafficking and all forms of violence and torture against children) were also prioritised. Many ideas addressed more than one SDGs concurrently. With this post-hoc analysis, there was more diversity in the SDG targets addressed by the highest ranked ideas, whereas the ideas that were ranked lower either did not address an SDG or often related to SDG 3.8 (achieving UHC, including quality of care) and usually appealed to the improvement of the quality of care aspect of the target. It would be interesting to conduct another CHNRI exercise to specifically explore uses of crowdsourcing for SDG targets.

The Millennium Development Goals (MDGs) were not achieved by many countries, and at the launch of the SDGs, no country was meeting all the health-related SDGs [26]. Many argue that the SDGs are more ambitious, and now the world is facing greater challenges as populations are becoming rapidly displaced. Indeed, one in 113 people are displaced according to recent statistics, which is the highest since the UNHCR began keeping records [27]. We cannot think of health without thinking of risk factors that affect almost 1% of the world’s population. Innovative solutions, such as crowdsourcing, may help achieve these goals. Indeed, the ideas generated address many of the SDGs. It would be beneficial to conduct an additional exercise exploring how crowdsourcing could address SDGs, specifically.

### Limitations

There were many more experts in global health compared to crowdsourcing who scored (n = 38 vs n = 15, respectively) which may be reflected in the scores. For example, as mentioned earlier, the idea that involved use of machine learning scored low in technological possibility although machine learning is used; this may be reflective of a predominantly global health expert base not being aware of advances in computer science. Conversely, experts in crowdsourcing may not have been aware of the needs in global health. This was indeed reflected in some of the ideas submitted which may not have responded to global health priorities. In future, it may be beneficial to conduct a workshop or a meeting to have both sides updated on advances in each other’s fields.

We did not provide a comprehensive definition of crowdsourcing prior to the commencement of the exercise. An earlier review found that the definition of crowdsourcing is contested and we felt this might confuse experts or dissuade their participation [28]. However, not supplying this definition has resulted in ideas that may fall into the realms of mHealth but not satisfying the definition of crowdsourcing (ie, no open call) [28], use contentious forms of crowdsourcing, including data mining, or do not articulate how exactly crowdsourcing could be used (eg, stating ‘use crowdsourcing to’ without giving specifics).

Some similar ideas scored quite differently, both in the overall ranking and in individual criteria. For example, an idea asking to use community members to generate vital registration data scored in the bottom 15 (#225) despite two of the top 15 ideas referring to using crowdsourcing to generate vital registration data. This idea relied on community members to come informed through a mass media campaign and provide data rather than religious leaders or CHWs, which could explain the difference in scores. Its lowest scores were in feasibility, perhaps not believing that end-users would have the necessary technology and in cost, time and innovation, perhaps believing that it would take too long to implement with a mass media campaign and that it could be expensive. Thus, the differences in scores of similar ideas could be a sign that experts were overloaded with ideas to score or, contrarily, could demonstrate that minor differences are truly important. There were also examples of similar ideas scoring almost the same; for example, #69 and 70, which asked to use public and adolescent feedback, respectively, to shape messaging for public health campaigns.

Finally, this exercise had a lower response rate than most previous CHNRI exercises. This is likely a result of a combination of factors, including less follow-up from our side, less familiarity and perhaps less commitment in the primary career areas to the content of the exercise, and a larger number of ideas which resulted in scorer fatigue. This could have also resulted in selection bias. Furthermore, we received feedback that not all criteria were relevant to each idea, which could have also contributed to poorer response. Initially, we planned to eliminate ideas where there were high proportions of blank scores across criteria (indicating the ideas were ‘unscoreable’ against the criteria); however, we noticed that this was not the case in any of the high scoring ideas.

## CONCLUSION

With 90% of the world’s population carrying a mobile phone [29], the potential for involving laypeople in health decisions or health reporting through mHealth technology is enormous. While crowdsourcing has been used in global health in pilot studies for diagnostics and in platforms in humanitarian settings already, it is not being used to its full potential [30]. This exercise is the first systematic, transparent assessment of the potential of crowdsourcing in global health and conflict settings. Its results can help future researchers, funding agencies, donors and policy makers choose which would be most appropriate and promising for their goals. Crowdsourcing is low-cost, rapid, and can complement traditional data collection; these qualities make it especially advantageous in global health, conflict or humanitarian settings.

## References

[1] Galton F (1949). Vox populi.. Nature.

[2] Howe J. (2006). The rise of crowdsourcing.. Wired Magazine.

[3] Estellés-Arolas E, González-Ladrón-de-Guevara F (2012). Towards an integrated crowdsourcing definition.. J Inf Sci.

[4] Ranard BL, Ha YP, Meisel ZF, Asch DA, Hill SS, Becker LB (2014). Crowdsourcing-harnessing the masses to advance health and medicine, a systematic review.. J Gen Intern Med.

[5] Wazny K (2018). Applications of crowdsourcing in health: an overview.. J Glob Health.

[6] Mavandadi S, Dimitrov S, Feng S, Yu F, Sikora U, Yaglidere O (2012). Distributed medical image analysis and diagnosis through crowd-sourced games: a malaria case study.. PLOS ONE.

[7] Chunara R, Chhaya V, Bane S, Mekaru SR, Chan EH, Freifeld CC (2012). Online reporting for malaria surveillance using micro-monetary incentives, in urban India 2010-2011.. Malar J.

[8] Shapiro DN, Chandler J, Mueller PA (2013). Using mechanical turk to study clinical populations.. Clin Psychol Sci.

[9] Pedersen J, Kocsis D, Tripathi A, Tarrell A, Weerakoon A, Tahmasbi N, et al. Conceptual foundations of crowdsourcing: a review of IS research. System Sciences (HICSS), 2013 46th Hawaii International Conference on; 2013: IEEE.

[10] Xie Y, Chen Z, Cheng Y, Zhang K, Agrawal A, Liao W-K, et al. Detecting and tracking disease outbreaks by mining social media data. Proceedings of the Twenty-Third international joint conference on Artificial Intelligence; 2013: AAAI Press.

[11] Ramanathan A, Pullum L, Steed CA, Quinn SS, Chennubhotla CS, Parker T. Integrating heterogeneous health care datasets and visual analytics for disease bio-surveillance and dynamics. 4th Wkshp on Integrative Text & Visual Analytics, IEEE Conf on Visual Analytics (VAST), Atlanta; 2013.

[12] Monitoring maternal, newborn and child health: understanding key progress indicators. Geneva, Switzerland: UNICEF, World Health Organisation; 2011.

[13] Ross JS, Mocanu M, Lampropulos JF, Tse T, Krumholz HM (2013). Time to Publication Among Completed Clinical Trials.. JAMA Intern Med.

[14] Rudan I, Gibson JL, Ameratunga S, El Arifeen S, Bhutta ZA, Black M (2008). Setting priorities in global child health research investments: guidelines for implementation of CHNRI method.. Croat Med J.

[15] Rudan I, Yoshida S, Chan KY, Sridhar D, Wazny K, Nair H (2017). Setting health research priorities using the CHNRI method: VII. A review of the first 50 applications of the CHNRI method.. J Glob Health.

[16] Bhutta ZA, Yakoob MY, Lawn JE, Rizvi A, Friberg IK, Weissman E (2011). Stillbirths: what difference can we make and at what cost?. Lancet.

[17] Dua T, Tomlinson M, Tablante E, Britto P, Yousfzai A, Daelmans B (2016). Global research priorities to accelerate early child development in the sustainable development era.. Lancet Glob Health.

[18] Kennedy HP, Yoshida S, Costello A, Declercq E, Dias MA, Duff E (2016). Asking different questions: research priorities to improve the quality of care for every woman, every child.. Lancet Glob Health.

[19] Bhutta ZA, Das JK, Walker N, Rizvi A, Campbell H, Rudan I (2013). Interventions to address deaths from childhood pneumonia and diarrhoea equitably: what works and at what cost?. Lancet.

[20] Walley J, Lawn JE, Tinker A, de Francisco A, Chopra M, Rudan I (2008). Primary health care: making Alma-Ata a reality.. Lancet.

[21] Kamajian SD (2015). How Crowdsourcing & Crowdfunding Are Fueling Health Care Innovation.. Osteopathic Family Physician..

[22] Philips M, Markham A (2014). Ebola: a failure of international collective action.. Lancet.

[23] (2014). Ebola: a failure of international collective action.. Lancet.

[24] Freifeld CC, Chunara R, Mekaru SR, Chan EH, Kass-Hout T, Iacucci AA (2010). Participatory Epidemiology: Use of Mobile Phones for Community-Based Health Reporting.. PLoS Med.

[25] King S., personal email communication to Wazny K, 2017.

[26] (2016). Measuring the health-related Sustainable Development Goals in 188 countries: a baseline analysis from the Global Burden of Disease Study 2015.. Lancet.

[27] Global Trends in Forced Displacement in. 2015. Geneva, Switzerland: United Nations High Commission for Refugees; 2016.

[28] Wazny K (2017). ‘Crowdsourcing’ ten years in: a review.. J Glob Health.

[29] Tamrat T, Kachnowski S (2012). Special Delivery: An analysis of mHealth in maternal and newborn health programs and their outcomes around the world.. Matern Child Health J.

[30] Zhao YX, Zhu QH (2014). Evaluation on crowdsourcing research: Current status and future direction.. Inf Syst Front.

