# Supplementary material for: Identifying potential uses of crowdsourcing in global health, conflict, and humanitarian settings: an adapted CHNRI (Child Health and Nutrition Initiative) exercise
Source: J Glob Health. 2018 Nov 9;8(2):020704. doi: 10.7189/jogh.08.020704 (PMC6220355; doi:10.7189/jogh.08.020704)
Supplement: Online Supplementary Document [file jogh-08-020704-s001.pdf]

Online Supplementary Document

Wazny et al. Identifying potential uses of crowdsourcing in global health, conflict, and humanitarian settings: an adapted CHNRI (Child Health and Nutrition Initiative) exercise

J Glob Health 2018;8:020704

Table S1. *List of scorers, affiliations:*

Name	Affiliation(s)
Durre Samin Akram	• Health Education and Literacy Program<br>• Aga Khan University of Health Sciences
Moazzam Ali	• World Health Organisation
Hani Atrash	• School of Public Health, Emory University
Diego Bassani	• Department of Paediatrics, the Hospital for Sick Children<br>• Dalla Lana School of Public Health, University of Toronto
Katherine Ba-thike	• University of Medicine, Yangon
Shinjini Bhatnagar	• Paediatric Biology Centre, Translational Health Science and Technology Institute, Government of India
Sohinee Bhattacharya	• Dugald Baird Centre for Research on Women's Health, Aberdeen Maternity Hospital
Zulfiqar A. Bhutta	• Centre for Global Child Health, the Hospital for Sick Children
Robert E. Black	• Institute for International Programs, Johns Hopkins School of Public Health
Josip Car	• Centre for Population Health Sciences, Lee Kong Chian School of Medicine, Nanyang Technological University
Kit Yee Chan	• Centre for Global Health Research, Usher Institute of Population Health Sciences and Informatics, University of Edinburgh
Stanley Chitekwe	• UNICEF Nepal

Joseph de Graft-Johnson	• Department of Global Health, Save the Children
Theresa Diaz	• UNICEF
Adegoke Falade	• College of Medicine, University of Ibadan<br>• University College Hopsital, Ibadan
Christa Fischer-Walker	• Johns Hopkins Bloomberg School of Public Health
Lynne Garcia	• LSG & Associates
Melissa Gladstone	• Department of Women and Children's Health, Institute of Translational Medicine, University of Liverpool<br>• Alder Hey Children's NHS Foundation Trust
Adenike Grange	• International Pediatric Association
David Hamer	• Center for Global Health Development and Department of Global Health, Boston University School of Public Health, Section of Infectious Diseases<br>• Fiedman school of Nutrition Science and Policy, Tufts University
Richard Heller	• People's Open Access Education Initiative
Sue Horton	• School of Public Health and Health Systems and Department of Economics, University of Waterloo
Tanvir Huda	• Sydney School of Public Health, University of Sydney<br>• Maternal and Child Health Division, icddr,b
Cholpon Imanalieva	• UNICEF Kyrgyzstan
Karin Kallandar	• Malaria Consortium<br>• Karolinska Institutet
Taha Kass-Hout	• Humanitarian Tracker
Dyness Kasungami	• John Snow Incorporated
Miguel Luengo-Oroz	• Biomedical Image Technologies, ETSI Telecomunicación, Universidad Politécnica de Madrid<br>• CIBER-BBN
Margaret Manley	• Centre for Global Child Health, the Hospital for Sick Children

Homero Martinez	• Micronutrient Initiative<br>• Hospital Infantil de Mexico
Sam Mavandadi	• University of California, Los Angeles
Golam Mothabbir	• Save the Children
Morkor Newman	• World Health Organisation
Angela Okolo	• Department of Pediatrics, Delta State University
David Osrin	• University College London Institute for Global Health
Taiwo Oyelade	• World Health Organisation
Bob Pattinson	• SAMRC/UP Maternal and Infant Health Care Strategies Unit, South Africa
Daniel Pope	• Department of Public Health and Policy, University of Liverpool
Barbara Prainsack	• Department of Global Health and Social Medicine, Kings College London
Ben Ranard	• Department of Medicine, Duke University School of Medicine
Mary-Elizabeth Reeve	• March of Dimes Foundation
Anna Roca	• Medical Research Council
Jakob Rogstadius	• Scania CV AB
H.P.S. Sachdev	• Sitaram Bhartia Institute of Science and Research
Salim Sadruddin	• World Health Organisation
Julio Saez-Rodriguez	• Faculty of Medicine, Joint Research Center for Computation Biolomedicine, RWTH Aachen University<br>• European Molecular Biology Laboratory, European Bioinformatics Institute, Wellcome Trust Genome Campus
Mathuram Santosham	• American International Health and Pediatrics, Johns Hopkins University
Budhi Setiawan	• UNICEF Myanmar
Philip Sherman	• Canadian Institutes of Health Research
Lorainne Tudor Car	• Lee Kong Chian School of Medicine, Nanyang Technological

	University<br>• Department of Public Health, Faculty of Mediicne, Imperial College London<br>• Department of Global Health and Population, Harvard T.H. Chan School of Public Health
Emorn Udomkesmalee	• Institute of Nutrition, Mahidol University
Kerri Wazny	• Centre for Global Health Research, Usher Institute for Population Health Sciences and Informatics, University of Edinburgh
Aisha Yousafzai	• Department of Global Health and Population, Harvard T.H. Chan School of Public Health

Box S1. *Further description of CHNRI calculations*

Scorers were asked to respond to the criteria, which are phrased as yes/no questions, by filling in the blanks as yes (a score of '1'), no (a score of '0' or 'not sure' (a score of '0.5). If a scorer felt that the criteria did not suit the idea or he/she was not informed, the scorer was to leave the cell blank. In calculating the scores for each idea, first within-criterion scores were calculated (which are displayed within the tables). We excluded blank cells from the denominator (so only cells that were scored yes, no, or unsure were used to calculate a score). Initially, we had intended to exclude ideas that had a high proportion of blank scores across criteria; however, these were not found in any high-scoring ideas and therefore would have had little impact on the actual results.

Once the within-criteria scores were calculated, we calculated an average across within-criteria scores to determine the Research Priority Score (RPS). The RPS score determines the relative ranking of the ideas (with the highest RPS indicating the top-scoring idea).

The Average Expert Agreement (AEA) is an indication of collective optimism across the crowd scoring. It is calculated by taking the mode (most frequently scored response) and dividing it by the total number of responses, again excluding blank cells. We use the AEA rather than a Kappa score because, although Kappa is designed to show statistical significance, since there are only three possible responses and a large number of scorers, there would be no way to show significance. The AEA is also thought to be more intuitive to interpret, being on a scale of 100.

Table S2. *Full list of ideas*

RANK	RESEARCH QUESTION	Category	C1: Tech Poss	C2: Feas	C3: Issues of Use	C4: Scale	C5: Impact	C6: Equity	C7: Inn	RPS	AEA
1	Use large crowds of health workers to rapidly share information about disease outbreak to ensure a more rapid global response (such as in the recent West African Ebola outbreak).	Epidemic responses	0.87	0.77	0.90	0.79	0.86	0.72	0.73	0.80	0.73
2	Create an online marketplace where health workers in remote villages could take a picture of a skin condition, for example, using their cell phones and post them to an internet accessible site where dermatologists from around the world could volunteer to 'read' them and give an opinion on their diagnosis. These diagnoses could then be returned to the healthcare worker by SMS.	Diagnostics	0.90	0.69	0.89	0.77	0.62	0.74	0.77	0.77	0.67
3	Use churches/mosques, village leaders or community health workers to take the lead in promoting developing a vital registration system.	Data generation	0.88	0.71	0.94	0.67	0.79	0.64	0.74	0.76	0.70
4	Use SMS for notification of birth and deaths in real-time, *especially in hard-to-reach areas and during conflict/natural disaster*.	Data generation	0.87	0.83	0.88	0.78	0.70	0.56	0.70	0.76	0.68
5	Ask CHWs to report demographic features of their communities, such as the number of householders with a female head and number of community meetings within a month.	Data generation	0.95	0.68	0.84	0.71	0.73	0.58	0.81	0.76	0.70

RANK	RESEARCH QUESTION	Category	C1: Tech Poss	C2: Feas	C3: Issues of Use	C4: Scale	C5: Impact	C6: Equity	C7: Inn	RPS	AEA
6	Crowdsource pathology images for research studies.	*Research	0.88	0.64	0.87	0.68	0.76	0.55	0.85	0.75	0.68
7	Ask large crowds of health workers to suggest ways to make labour and delivery safer.	Problem-solving	0.89	0.91	0.90	0.60	0.61	0.49	0.83	0.75	0.66
8	Harness the knowledge of CHWs, field workers and stakeholders outside the health arena to solve challenges associated with implementation and scaling-up of programs.	Problem-solving	0.89	0.66	0.98	0.69	0.66	0.60	0.76	0.75	0.69
9	Use crowdsourcing to map outbreaks (e.g. measles), immunisation dark spots, access local transportation for emergencies, identification of medical resources, etc.	Epidemic responses	0.79	0.68	0.88	0.71	0.75	0.69	0.73	0.75	0.60
10	Use large crowds of health workers to report on every instance when they witness a maternal or child death, the cause of that death and if the death could have been prevented.	Data generation	0.98	0.87	0.82	0.61	0.69	0.59	0.61	0.74	0.65
11	Have experts contribute voluntarily to develop freely available online training courses (or get national funding to deliver printed materials) that teach documented, high-impact skills, which are in short supply. Design the courses with the intent to let members of communities in need learn to solve problems in their own communities. These educational materials should likely be broad in scope, including basic management or business practices to ensure that the service can be kept running in a sustainable way.	Education	0.88	0.67	0.84	0.71	0.72	0.62	0.74	0.74	0.64

RANK	RESEARCH QUESTION	Category	C1: Tech Poss	C2: Feas	C3: Issues of Use	C4: Scale	C5: Impact	C6: Equity	C7: Inn	RPS	AEA
12	Epidemic identification in remote environments through collection and analysis of crowd-sourced detection Healthcare workers (and possibly ordinary individuals) are asked to report new/unusual diseases using mobile phones (e.g. taking photographs) to central server which then compiles and analyzes the data.	Epidemic responses	0.83	0.58	0.85	0.74	0.83	0.67	0.66	0.74	0.64
13	Ask large crowds of global health workers to report through text messages on hours/days spent on travel and conferences/workshops in a specified period and to suggest how they could have learned/contributed without travel in order to find solutions to reduce the carbon footprint and increase efficiency in global health.	Problem-solving	0.86	0.79	0.82	0.68	0.64	0.64	0.74	0.74	0.63
14	Use large crowds to assess community demand. Encourage communities to share by SMS a service they believe needs to be in place to support health and development in their community as an opportunity to explore demand-side push for services at a larger scale.	Problem-solving	0.94	0.73	0.90	0.68	0.67	0.53	0.69	0.73	0.62
15	Use large crowds of diagnostic tool makers and other scientists to generate new, accurate and reliable, low cost, non-invasive, scalable methods for measuring nutrition-relavant issues such as gestational age and size at birth, low birth weight, length or height, exclusive breastfeeding, usual dietary intake and other forms of nutritional status (e.g. multiple micronutrient deficiencies).	Novel discoveries	0.78	0.68	0.92	0.74	0.67	0.66	0.69	0.73	0.66

RANK	RESEARCH QUESTION	Category	C1: Tech Poss	C2: Feas	C3: Issues of Use	C4: Scale	C5: Impact	C6: Equity	C7: Inn	RPS	AEA
16	Ask men who have sex with men to anonymously report outbreaks of diseases within their circles, such as sexually transmitted intestinal infections (shigellosis, amebiases, etc.).	Data generation	0.86	0.57	0.96	0.72	0.65	0.57	0.79	0.73	0.68
17	Have HCWs post problems needing help and ask for offer/suggestions to solve these problems to improve quality of care, also route problems to appropriate bodies. Focus outputs from these exercises on key points that influence policy/decision making (e.g. public seats on government agencies to ensure registration and standardisation bodies aware so they are taken seriously).	Problem-solving	0.83	0.91	0.86	0.68	0.60	0.54	0.70	0.73	0.64
18	Use large crowds of community health workers to conduct community surveys and report findings (baseline vaccination coverage, nutritional status, for instance), to be used by the health program to target and implement specific interventions.	Data generation	0.90	0.72	0.88	0.60	0.65	0.69	0.68	0.73	0.65
19	Ask health case workers to identify barriers to provision of better care and treatment to patients via SMS.	Problem-solving	0.89	0.74	0.87	0.71	0.67	0.59	0.63	0.73	0.64
20	Ask the general population to identify the top 3 health issues that they visualize around them presently. Create a map of the health issues submitted. This could be used to find people or organizations interested in tackling these issues within a close vicinity.	Problem-solving	0.88	0.86	0.93	0.63	0.57	0.57	0.63	0.73	0.65
21	Gather data on disease spread -- health workers reporting on diagnoses through their work day (tracking spread of disease and trends).	Data generation	0.87	0.67	0.92	0.66	0.62	0.66	0.68	0.73	0.63

RANK	RESEARCH QUESTION	Category	C1: Tech Poss	C2: Feas	C3: Issues of Use	C4: Scale	C5: Impact	C6: Equity	C7: Inn	RPS	AEA
22	Create a platform that can crowdsource diverse skills (incl. engineers, statisticians, computer scientists, psychologists, markteting professionals, scientists and HCWs) to solve bottlenecks that arise in the healthcare value chain, ranging from medical supply chain and logistics for vaccines, essential medicines, demand creation to quality of care.	Problem-solving	0.83	0.59	0.87	0.71	0.73	0.62	0.72	0.72	0.63
23	Get feedback from affected people in a crisis regarding the humanitarian response, what were their needs, were they met, etc.	Epidemic responses	0.88	0.70	0.84	0.65	0.54	0.73	0.73	0.72	0.63
24	Have staff report via SMS the last time they were trained on a specified topic.	Education	0.82	0.83	0.87	0.67	0.60	0.62	0.65	0.72	0.65
25	Use large crowds of school teachers and/or community leaders to report on gender-based violence (via SMS) either in the home or in public spaces, as an alternative to surveys.	Data generation	0.89	0.84	0.88	0.71	0.68	0.48	0.58	0.72	0.65
26	Ask families and individuals to contribute their perspectives on experiences of ill health, its causes and of seeking and obtaining treatment and care through SMS. Offer airtime as an incentive to respond.	Problem-solving	0.90	0.67	0.90	0.72	0.58	0.59	0.70	0.72	0.65

RANK	RESEARCH QUESTION	Category	C1: Tech Poss	C2: Feas	C3: Issues of Use	C4: Scale	C5: Impact	C6: Equity	C7: Inn	RPS	AEA
27	Create a "Health Advisor" where every aspect of improper treatment, including being made to pay for services meant to be free, being treated poorly, not being given appropriate medicines, essential medicines being unavailable, equipment being unavailable or in disrepair, etc., are reported to a public accountability site via SMS by patients of health centres. The public accountability site will gather reports and release findings every 3 months. After findings are made public, teams are sent to health centres to re-train them on challenges found in the report.	Data generation	0.91	0.72	0.90	0.67	0.58	0.60	0.67	0.72	0.66
28	Ask large crowds of teenagers to suggest ways to adopt healthy adolescent behaviours or discourage unhealthy behaviours, such as preventing adolescents from starting to smoke.	Problem-solving	0.82	0.69	0.89	0.65	0.65	0.66	0.69	0.72	0.62
29	Ask large crowds of community health workers to report prospectively on maternal and perinatal mortality at the community-level and probable causes.	Data generation	0.84	0.72	0.85	0.77	0.68	0.49	0.67	0.72	0.66
30	Use crowdsourcing to determine prevalence and incidence data in LMICs for disease in which we have poor data, such as mental health or types of physical disability by asking CHWs for the numbers of people in their communities with these specific conditions.	Data generation	0.73	0.82	0.88	0.58	0.59	0.64	0.78	0.72	0.62
31	Ask patients with specific illnesses how they cope, obstacles and barriers as well as facilitators to achieving their treatment goals.	Problem-solving	0.92	0.89	0.82	0.46	0.56	0.64	0.71	0.72	0.63

RANK	RESEARCH QUESTION	Category	C1: Tech Poss	C2: Feas	C3: Issues of Use	C4: Scale	C5: Impact	C6: Equity	C7: Inn	RPS	AEA
32	Ask large crowds of local health workers involved in vaccination to capture parents' concerns about vaccines and reasons for vaccine refusal at the point of care. This will help identify the reason and location of refusal in real-time.	Problem-solving	0.85	0.66	0.86	0.61	0.63	0.73	0.68	0.72	0.66
33	Could crowd sourcing be used to diffuse conflicts and de-escalate tensions in a trouble spot? This may be a simple (managed) social media exercise but more though is needed.	Problem-solving	0.86	0.68	0.81	0.58	0.73	0.63	0.71	0.71	0.62
34	Use large crowds of local health workers to report on availability and utilization of specific national or regional health intervention guidelines via mobile text messaging.	Data generation	0.90	0.70	0.87	0.58	0.63	0.60	0.70	0.71	0.64
35	Use a Facebook page or Twitter to generate ideas for research (the CHNRI method) but done via Facebook or Twitter.	Problem-solving	0.81	0.78	0.87	0.66	0.53	0.65	0.68	0.71	0.59
36	Use mobile phone feedback to rapidly map disease outbreaks.	Data generation	0.78	0.72	0.73	0.77	0.76	0.58	0.63	0.71	0.63
37	Use large crowds of pregnant mothers to report by SMS every instance of disrespectful care, including type of disrespect received, during antenatal care or childbirth to gather data and monitor in real-time levels and changes in service providers' behaviours towards pregnant women and recently delivered mothers.	Data generation	0.82	0.79	0.87	0.59	0.62	0.61	0.67	0.71	0.62
38	Use mobile phone feedback to map disasters by scale, location and to assess needs	Epidemic responses	0.73	0.74	0.84	0.68	0.75	0.54	0.67	0.71	0.61

RANK	RESEARCH QUESTION	Category	C1: Tech Poss	C2: Feas	C3: Issues of Use	C4: Scale	C5: Impact	C6: Equity	C7: Inn	RPS	AEA
39	Use crowdsourcing (expanded version, including health professionals but also engineers, marketing professionals, economists, psychologists, computer scientists, etc.) to identify creative solutions for very specific and well-defined global health issues, bottlenecks or roadblocks. An example of a bottleneck would be how to increase coverage of a given vaccine or how to increase the prevalence of exclusive breastfeeding. The problems should be defined in light of the best possible results achieved by best practices and should try to address the possible strategies to get past the best results and improve on them.	Problem-solving	0.90	0.76	0.90	0.71	0.52	0.54	0.60	0.70	0.63
40	Ask crowds of individuals to report on key performance indicators for HCWs (for example, for reproductive health, ask for instances of HCWs discussing readiness for pregnancy, abstinence of alcohol as a means to prevent fetal alcohol syndrome, etc.).	Problem-solving	0.78	0.69	0.83	0.70	0.67	0.74	0.53	0.70	0.60
41	Create a responsibly moderated wiki-type appraoch that could be run for a set time (3-12 months) where the informed/intelligent lay population is provided with key facts and references about priority problems and what is known about impacts, costs, strengths and weaknesses of a range of preventive and treatment options for a specified problem. The result will be where the knowledge gaps are/appear to be and what needs to be addressed as global health priorities.	Problem-solving	0.85	0.67	0.81	0.51	0.64	0.66	0.79	0.70	0.62

RANK	RESEARCH QUESTION	Category	C1: Tech Poss	C2: Feas	C3: Issues of Use	C4: Scale	C5: Impact	C6: Equity	C7: Inn	RPS	AEA
42	Ask large crowds of CHWs to report unusual cases and infectious diseases of public health concern for early detection of outbreaks (cholera, malaria, haemorrhagic fever, contact tracing in Ebola epidemic, etc.).	Epidemic responses	0.63	0.72	0.93	0.65	0.68	0.63	0.70	0.70	0.58
43	Crowdsource service delivery information such as availability of healthcare at given institutions.	Data generation	0.85	0.68	0.85	0.51	0.68	0.63	0.73	0.70	0.63
44	Ask HCWs what they use as a first-line antiobiotics treatment for various childhood diseases.	Data generation	0.89	0.78	0.81	0.69	0.58	0.53	0.63	0.70	0.63
45	Ask large crowds of health workers to report by SMS or a mobile phone open-source application of the complications witnessed when treating or referring patients, such as obstetric complications, in real-time.	Data generation	0.90	0.75	0.84	0.52	0.63	0.55	0.73	0.70	0.61
46	Ask large crowds of local health workers to report on availability of cadres of health care providers in underserved and hard-to-reach areas via text messaging.	Data generation	0.83	0.76	0.80	0.67	0.56	0.58	0.71	0.70	0.59
47	Have expert graders assess doctor's skills remotely to improve on procedural and technical skills and assist with improvement.	Education	0.89	0.52	0.84	0.63	0.69	0.66	0.65	0.70	0.61
48	Use crowdsourcing as a rapid way to conduct formative research to better understand behavioural determinants, i.e. factors that faciliate or inhibit adoption of healthy practices. For example, 'why don't people commonly bring sick children to X, what need to happy to increase Y or decrease Z.'	*Research	0.76	0.74	0.82	0.65	0.63	0.66	0.64	0.70	0.60

RANK	RESEARCH QUESTION	Category	C1: Tech Poss	C2: Feas	C3: Issues of Use	C4: Scale	C5: Impact	C6: Equity	C7: Inn	RPS	AEA
49	Use crowdsourcing to facilitate and scale-up the traditional laborious "positive deviant inquiry" to identify rare examples of "social success" against a backdrop of generalized failure AND identify clues that might explain the success.	*Research	0.86	0.76	0.81	0.57	0.56	0.57	0.74	0.70	0.63
50	Ask children in national school competitions to come up with the best ideas for changing a certain aspect of global health (i.e. a technology project for all secondary school classes which can be won).	Problem-solving	0.76	0.80	0.90	0.55	0.56	0.63	0.66	0.70	0.63
51	Use big data from Internet sites like Facebook, WhatsApp, Instagram pictures and Google searches to study trending health information search words and phrases in order to predict disease outbreaks in communities in order to target health education.	Data generation	0.88	0.66	0.91	0.61	0.56	0.51	0.73	0.69	0.62
52	Crowdsource health workers to come up with innovative solutions to accelerate eradication of polio in countries like Pakistan, Afghanistan and Northern Nigeria through SMS surveys.	Epidemic responses	0.82	0.77	0.92	0.65	0.58	0.47	0.64	0.69	0.62
53	Have patients visiting health centres report what was missing from their visit/care.	Problem-solving	0.75	0.75	0.81	0.68	0.61	0.69	0.55	0.69	0.60
54	Polling large groups of health workers in LMICs at different levels on needs and ideas for engineered devices that would most impact the populations they work with.	Problem-solving	0.60	0.57	0.90	0.77	0.66	0.68	0.66	0.69	0.62
55	Use a YouTube video to show a typical encounter of a care taker in a health clinic and ask both care takers and health providers to provide feedback on the site as to what could have been done better or what was done well.	Problem-solving	0.92	0.66	0.78	0.63	0.57	0.58	0.70	0.69	0.59

RANK	RESEARCH QUESTION	Category	C1: Tech Poss	C2: Feas	C3: Issues of Use	C4: Scale	C5: Impact	C6: Equity	C7: Inn	RPS	AEA
56	Use mobile phones for periodic morbidity and mortality surveillance, addressing the lack of morbidity and mortality data. An example would be, say, at a health facility doing deliveries for for communities midwives or TBAs, text 1 for each healthy birth, - for a still birth, 2 for sick but alive newborn.	Data generation	0.73	0.79	0.84	0.61	0.67	0.57	0.60	0.69	0.58
57	Ask people if programs address the sexual health concerns of difference societal groups (i.e. families, youth, high-risk groups) and whether people acknowledge that this is an important component of their health. Ask people what role mass media play in this regard and survey the media to see if they are aware of their responsibilities.	Problem-solving	0.87	0.73	0.86	0.56	0.58	0.57	0.66	0.69	0.61
58	Poll large crowds of health workers by phone or email to learn what motivates or demotivates them. Poll them on their best day and worst day at work in a given period, compare between levels of care (community, first-level, referral) and types of health workers (CHWs, nurse, doctor, etc.).	Problem-solving	0.73	0.57	0.78	0.64	0.73	0.63	0.73	0.69	0.58
59	Analyse some relevant data source (e.g. patient journals, interview patients or nurses or use SMS surveys) to identify frequent health problems that members of community experience. From these, identify problems that can easily be handled at home without professional healthcare and produce a booklet that gives adcise for self-help and distribute it freely to patients at health centres.	Problem-solving	0.83	0.66	0.79	0.59	0.60	0.70	0.64	0.69	0.63
60	Use crowdsourcing to come up with novel solutions to drive evidence to policy.	Novel discoveries	0.70	0.77	0.90	0.56	0.53	0.58	0.78	0.69	0.59

RANK	RESEARCH QUESTION	Category	C1: Tech Poss	C2: Feas	C3: Issues of Use	C4: Scale	C5: Impact	C6: Equity	C7: Inn	RPS	AEA
61	Ask large crowds of parents to report successful tips for healthy child practices, including early initiation and exclusive breastfeeding and to share encouragement for other parents to immunize their children against vaccine preventable diseases.	Problem-solving	0.82	0.71	0.78	0.73	0.60	0.55	0.62	0.69	0.59
62	Ask large crowds of local health works to generate locally appropriate, creative approaches on how to promote preventive health behaviours and sustain them (i.e. dietary, WASH, ECD).	Problem-solving	0.77	0.79	0.78	0.56	0.65	0.61	0.66	0.69	0.60
63	Assemble crowdsourcing outputs to give trends that track over time.	Data generation	0.79	0.83	0.76	0.66	0.65	0.54	0.56	0.69	0.60
64	At conference registrations, as a requirement for registration, have a questionnaire on what the registrant has done to improve quality of services or opinions on how to improve quality of services. Do this at multiple conferences.	Problem-solving	0.74	0.76	0.84	0.67	0.57	0.61	0.61	0.69	0.59
65	Ask mothers around the world to use their mobile phones to do a nominal group type process on what they see as the top three things that would make their child's health better.	Problem-solving	0.79	0.58	0.81	0.69	0.64	0.56	0.73	0.69	0.60
66	Using large crowds of youth and innovators, make a global contest to improve the uptake of ORS and zinc for diarrhoa treatment in children: this can be on presentation, taste packaging and way to market ORS and zinc wider.	Problem-solving	0.83	0.80	0.86	0.56	0.54	0.57	0.64	0.69	0.59

RANK	RESEARCH QUESTION	Category	C1: Tech Poss	C2: Feas	C3: Issues of Use	C4: Scale	C5: Impact	C6: Equity	C7: Inn	RPS	AEA
67	Ask a large crowd of national and regional program managers and community health worker supervisors to generate ideas on supervision strategies (including clinical supervision) which has worked in their setting for conducting implementation research or piloting in other settings.	Problem-solving	0.75	0.74	0.91	0.68	0.58	0.57	0.57	0.69	0.59
68	Have large crowds of frontline health workers use SMS to report on training and skill development needs in order for program managers to determine the capacity building needs of health workers.	Education	0.76	0.72	0.82	0.63	0.61	0.64	0.60	0.69	0.61
69	Ask for feedback from the general population on key health/nutrition messaging in different contexts, including how to 'sell' a particular message.	Problem-solving	0.90	0.72	0.84	0.60	0.55	0.49	0.69	0.68	0.63
70	Ask school children (grades 10, 11) for interesting ideas that can be used to popularize important public health messages (similar to industry's approach for coining logos and brand names for products, but with crowdsourcing).	Problem-solving	0.79	0.64	0.93	0.61	0.56	0.49	0.76	0.68	0.61
71	Ask large amounts of households whether they purchased drugs from the private or public sector and when they made their last purchase. Use this to understand the role of the private sector in the supply chain management for particular diseases (e.g. malaria) and to show availability and distribution of different drugs.	Data generation	0.92	0.90	0.83	0.66	0.50	0.42	0.54	0.68	0.61

RANK	RESEARCH QUESTION	Category	C1: Tech Poss	C2: Feas	C3: Issues of Use	C4: Scale	C5: Impact	C6: Equity	C7: Inn	RPS	AEA
72	Set up a website for adolescents to anonymously submit their questions and concerns. Questions would be reviewed by reproductive health education program planners involved in planning for the target population to identify common questions and concerns which would then be shared with local health workers and educators in a collaborative effort to design and implement reporductive health education that addresses the adolescents' concerns in a culturally appropriate manner. Some of the health education could be delivered electronically to maintain the greatest comfort level with the topic. A similar exercise could be conducted for other topics where there is stigmatisation or need for privacy.	Problem-solving	0.80	0.61	0.91	0.57	0.70	0.56	0.62	0.68	0.58
73	Use large groups of global health experts to generate anonymous feedback on the biggest mistakes they have made in their work, doing the same with NGO implementers and with field workers.	Problem-solving	0.75	0.72	0.80	0.57	0.64	0.59	0.69	0.68	0.55
74	Use crowdsourcing to annotate medical imaging, such as radiology and histopoathology slides.	Data generation	0.82	0.63	0.85	0.73	0.58	0.53	0.61	0.68	0.59
75	Ask large crowds of global health experts to report on identified evidence-based best practices for delivery of effective interventions via e-surveys.	Problem-solving	0.83	0.76	0.85	0.60	0.55	0.52	0.63	0.68	0.60
76	Ask large crowds of global health experts or health experts experienced in the specific region to generate ideas for a specific program design and for its evaluation to be reviewed but the implementor and used in the final design.	Problem-solving	0.85	0.70	0.87	0.56	0.60	0.54	0.63	0.68	0.61

RANK	RESEARCH QUESTION	Category	C1: Tech Poss	C2: Feas	C3: Issues of Use	C4: Scale	C5: Impact	C6: Equity	C7: Inn	RPS	AEA
77	Use large crowds of local community members to rapidly solve logistical problems, such as transporting rural pregnant women to a health facility timeously or transporting referrals (could team up with Uber and get them to take it on as CSR?).	Problem-solving	0.75	0.82	0.81	0.68	0.56	0.56	0.57	0.68	0.57
78	Use community members or village headman to report on the sanitation status of their villages (with the goal of targeting open defecation free zones).	Data generation	0.74	0.48	0.80	0.75	0.63	0.68	0.67	0.68	0.62
79	Use large crowds of CHWs to conduct mapping of various diseases (e.g. NTDs, malaria, polio, cholera, meningitis, top priority zoonotic diseases, etc.).	Data generation	0.67	0.63	0.76	0.69	0.71	0.60	0.68	0.68	0.60
80	Use inexpensive sensors in cell phones to collect personal data (with consent) to get added information about health events, can try to prevent these events based on signal changes.	Data generation	0.94	0.52	0.86	0.60	0.56	0.51	0.74	0.68	0.61
81	Ask large crowds of CHWs to report health issues (e.g. availability of and access to services for pregnant women) during acute and prolonged emergencies, including gradile stats and conflict areas.	Problem-solving	0.82	0.59	0.88	0.59	0.57	0.54	0.74	0.68	0.61
82	Use large crowds of global health experts to provide computational tools to solve some of the key questions in global health (i.e. predict the outbreak of pandemics from anything from the weather to telephone activity) -- this can be shaped in the form of collaborative competition, such as the DREAM challenges (www.dreamchallenges.org).	Problem-solving	0.85	0.83	0.82	0.52	0.58	0.53	0.60	0.68	0.59

RANK	RESEARCH QUESTION	Category	C1: Tech Poss	C2: Feas	C3: Issues of Use	C4: Scale	C5: Impact	C6: Equity	C7: Inn	RPS	AEA
83	Ask people their concerns regarding health and reproductive health and match these with local programs. Find if local programs are indeed addressing their concerns or if they are in fact ignoring people's concerns.	Problem-solving	0.79	0.74	0.73	0.64	0.63	0.64	0.55	0.67	0.55
84	Use large crowds of recently discharged patients to report progress in adherence to advice post-discharge during follow-up period, including Kangaroo Mother Care	Data generation	0.80	0.63	0.84	0.58	0.60	0.54	0.72	0.67	0.59
85	Use SMS and email for real-time reporting and monitoring of drug supply and availability in markets and/or of conterfeit drugs.	Data generation	0.65	0.70	0.87	0.68	0.73	0.48	0.60	0.67	0.60
86	Ask experts and men how family planning services can be integrated in primary health services in a way that ensures high quality and confidential service so men are effectively reached and able ot make informed decisions.	Problem-solving	0.89	0.85	0.86	0.60	0.55	0.42	0.55	0.67	0.62
87	Ask healthcare providers globally for suggestions for innovations.	Problem-solving	0.78	0.51	0.78	0.61	0.68	0.70	0.63	0.67	0.60
88	Equip CHWs in hard-to-reach areas with video-enables and picture-enabled smartphones and allow them to text photos and videos of difficult to diagnose cases to a supervisor.	Diagnostics	0.78	0.74	0.79	0.57	0.64	0.58	0.58	0.67	0.59
89	Use crowdsourcing to determine the distance patients had to travel to a health centre, how many staff were there when they arrived and what type of staff were there (i.e. were there any specialists, are there any specialists for mental health disorders).	Data generation	0.79	0.73	0.85	0.54	0.58	0.55	0.65	0.67	0.58

RANK	RESEARCH QUESTION	Category	C1: Tech Poss	C2: Feas	C3: Issues of Use	C4: Scale	C5: Impact	C6: Equity	C7: Inn	RPS	AEA
90	Equip health working who see paediatric cases to monitor all vaccination with adverse events following vaccination such as fever and rash. Use the data to visualise adverse events and illustrate that serious adverse events are extremely rare -- putting alongside visualizations on population-wide disease burden and vaccination impact.	Data generation	0.72	0.68	0.88	0.54	0.59	0.62	0.66	0.67	0.58
91	Use crowds of practitioners across different relevant disciplines to nutrition (e.g. health, agriculture, water, sanitation) to share experiences/come up with ideas of how to make their programmes more 'nutrition sensitive'	Problem-solving	0.90	0.68	0.90	0.48	0.47	0.51	0.76	0.67	0.61
92	Invite the public to send in their key priorities for health services in their area or the top three problems from their perspective where health services principles, guarantees or targets are not being met and draw a prize from the texts received.	Problem-solving	0.87	0.75	0.81	0.73	0.53	0.37	0.63	0.67	0.60
93	Ask clinicians and the public what will make clinicians follow protocols -- in other words, what will make them do the right thing?	Problem-solving	0.86	0.72	0.82	0.48	0.53	0.58	0.69	0.67	0.59
94	Create a repository of localised culturally relevant behaviour change messages around key critical practices.	Problem-solving	0.67	0.78	0.81	0.62	0.62	0.54	0.63	0.67	0.54
95	Ask clinicians and the public how the behaviour of clinicians (doctors and midwives) can be changed so they respect pregnant women's wishes and treat pregnant women with dignity.	Problem-solving	0.71	0.68	0.76	0.59	0.63	0.56	0.74	0.67	0.60

RANK	RESEARCH QUESTION	Category	C1: Tech Poss	C2: Feas	C3: Issues of Use	C4: Scale	C5: Impact	C6: Equity	C7: Inn	RPS	AEA
96	Use crowdsourcing to survey continuing medical education and investigate its distribution throughout a country in order to see how clustered it is in urban areas.	Education	0.79	0.81	0.75	0.60	0.61	0.50	0.61	0.67	0.58
97	Create a message board for clinicans to discuss their latest diagnostic or management dilemmas and allow free flow of information.	Diagnostics	0.85	0.62	0.84	0.65	0.59	0.54	0.58	0.67	0.58
98	Use the crowdsourcing tool to gauge HCWs understanding of a problem, i.e. failure to answer questions (e.g. 'no information' or 'cannot answer)' will give insight into the degree of understanding of the problem.	Data generation	0.87	0.87	0.76	0.64	0.54	0.40	0.59	0.67	0.59
99	Have health workers from multiple NGO-run programs text every time something did not go as planned or every failure to a repository. This will create a large repository of data on why large global health programs do not go as planned, down from the planning stages to those on the ground and from multiple programs from multiple NGOs. This will provide an opportunity to ensure challenges are recognized and addressed and programs are ultimately more effective.	Problem-solving	0.87	0.71	0.88	0.45	0.53	0.57	0.63	0.66	0.60
100	Have expert surgical graders assess surgical techniques remotely to improve on the training available to surgeons in LMICs.	Education	0.87	0.75	0.82	0.51	0.54	0.51	0.64	0.66	0.57
101	Ask CHWs to report whether their community has a community financing structure.	Data generation	0.88	0.72	0.79	0.59	0.49	0.60	0.57	0.66	0.60

RANK	RESEARCH QUESTION	Category	C1: Tech Poss	C2: Feas	C3: Issues of Use	C4: Scale	C5: Impact	C6: Equity	C7: Inn	RPS	AEA
102	Use large crowds of community leaders to report any corruption at any level in the health sector by SMS.	Data generation	0.76	0.72	0.89	0.59	0.51	0.51	0.66	0.66	0.60
103	Ask large crowds of local health workers to report reasons given to them by women why they do not come back to postpartum (interconception) care.	Problem-solving	0.74	0.76	0.70	0.54	0.58	0.69	0.63	0.66	0.58
104	Ask a large crowd of community members to report by SMS any instance of health discussion where data from the nearest health facility was used that they participated in to generate data to access community engagement.	Data generation	0.68	0.76	0.91	0.35	0.50	0.70	0.73	0.66	0.62
105	Create a method for trained citizens of all countries to register their expertise and willingness to help in the event of an urgent health need. When the need arises then the central office could send a message to the nearest registered users informing them of the need.	Problem-solving	0.83	0.73	0.86	0.62	0.56	0.43	0.58	0.66	0.59
106	Use large crowds of local community representatives, health committees and local government to use SMS to report on absenteeism of the health workers and health facilities or designated service delivery points on scheduled dates/times to get real-time information on the degree of health worker absenteeism and to help develop policies to address it.	Data generation	0.86	0.64	0.78	0.67	0.55	0.42	0.71	0.66	0.59
107	Use crowdsourcing to determine the waiting times at clinics and other health centres.	Data generation	0.73	0.52	0.81	0.69	0.63	0.66	0.58	0.66	0.60
108	Ask health care providers how communication between different healthcare services and levels of healthcare can be improved.	Problem-solving	0.80	0.78	0.79	0.58	0.58	0.46	0.60	0.66	0.59

RANK	RESEARCH QUESTION	Category	C1: Tech Poss	C2: Feas	C3: Issues of Use	C4: Scale	C5: Impact	C6: Equity	C7: Inn	RPS	AEA
109	Crowdsource health workers through SMS driver surveys to find out website they visit the most to search for information on disease diagnosis and management -- this could shed more light on potential application of web-based databases to strengthen quality of healthcare.	Problem-solving	0.88	0.68	0.89	0.54	0.58	0.49	0.54	0.66	0.60
110	Make available a common platform for routinely collected data from birth and death registries with standardized definitions and classifications.	Problem-solving	0.76	0.70	0.78	0.65	0.66	0.43	0.63	0.66	0.57
111	Ask crowds of experts in areas not strictly defined within the 'health' domain or outside the health domain (i.e. architecture, engineering, political studies) to generate a list of the top 10 most pressing health-related issues facing the world and how they could help solves those issues.	Problem-solving	0.74	0.57	0.81	0.65	0.54	0.63	0.67	0.66	0.57
112	Ask health workers to report the number of babies correctly resuscitated by trained and non-trained staff.	Data generation	0.78	0.80	0.90	0.58	0.51	0.51	0.52	0.66	0.57
113	Ask health care providers whether blood group testing is done prenatally and/or at deliver, with the intention of developing an understanding of the prevalence of hemolytic disease of the newborn in LMICs.	Data generation	0.83	0.82	0.86	0.54	0.44	0.46	0.64	0.66	0.60
114	Ask clinical experts for trends of use of certain medications, which is without a license (i.e. use of medication to treat aggressiveness in dementia -- currently no medication is licensed for this indication yet many compounds are used).	Data generation	0.77	0.77	0.81	0.59	0.51	0.55	0.58	0.65	0.56

RANK	RESEARCH QUESTION	Category	C1: Tech Poss	C2: Feas	C3: Issues of Use	C4: Scale	C5: Impact	C6: Equity	C7: Inn	RPS	AEA
115	Have local HCWs report via SMS when they were unable to adequately treat a condition/disease due to unavailability of proper medication.	Data generation	0.69	0.69	0.92	0.63	0.51	0.53	0.60	0.65	0.58
116	Ask relevant healthcare providers (i.e. obstetricians, neonatologists, physicians, nurses, community workers, midwives) how many deliveries occur anually, what proportion are in hospitals, clinics, community and other and which proportion of pregnancies are seen in antenatal clinics.	Data generation	0.78	0.67	0.77	0.56	0.65	0.57	0.57	0.65	0.56
117	Ask large crowds of patients and caregivers to report via mobile text messaging on factors affecting utilization of specific health services. For example, we could survey adolescents, or patients with chronic diseases specifically, such as HIV, to query obstacles to accessing information specific to their conditions such as information regarding prevention and management of adolescent health issues or reasons for missing medication. Then, data would be geocoded to sort problems (access, stigmatisation, lack of drugs at facilities, etc.).	Problem-solving	0.82	0.62	0.81	0.60	0.63	0.37	0.71	0.65	0.61
118	Use a large crowd of midwifery students to report by SMS any instance of neonatal asphyxia they obseve and the role they played to gather data to study student hands-on opportunities and its contribution to their learning.	Education	0.76	0.47	0.80	0.60	0.61	0.60	0.74	0.65	0.57
119	Use large crowds of school-teachers and/or community leaders to generate ideas on how to keep girls in school and/or delay early marriage.	Problem-solving	0.74	0.79	0.75	0.63	0.63	0.41	0.60	0.65	0.57

RANK	RESEARCH QUESTION	Category	C1: Tech Poss	C2: Feas	C3: Issues of Use	C4: Scale	C5: Impact	C6: Equity	C7: Inn	RPS	AEA
120	Ask large groups of mothers why they do not access health care services for preventive or curative care for their children by setting up a kiosk at a martketplace where they answer questions and get a prize for providing suggestions on what could be done to get them to use services.	Problem-solving	0.79	0.83	0.85	0.49	0.53	0.44	0.60	0.65	0.59
121	Rare diseases are often misdiagnosed as people have little experience with it, having people make their data vailable for rare diseases could help create data sets that are otherwise impossible.	Data generation	0.89	0.76	0.89	0.56	0.43	0.48	0.54	0.65	0.60
122	Ask large crowds of community health workers to report by mobile phone on activities conducted each day (persons visited, problems found, measures taken) and problems encountered (lack of meds, other supplies, patient referred, etc.) to be used by the health program to evaluate health worker progress, identify needs/problems and target interventions.	Problem-solving	0.76	0.79	0.80	0.44	0.49	0.61	0.66	0.65	0.57
123	Ask large crowds of private sector providers (or public sector providers, depending on the setting) to report on common treatments for routine childhood illnesses.	Data generation	0.73	0.66	0.77	0.51	0.57	0.62	0.68	0.65	0.56
124	Obtain real-time information from the lowest levels of service-delivery for a bottom-up approach in order to address obstacles, bottlenecks and inefficiencies to service delivery, have data tracked daily.	Problem-solving	0.77	0.65	0.76	0.60	0.59	0.54	0.63	0.65	0.54
125	Ask the crowd to collect diet and exercise data over a period of time then correlate to some outcome down the road.	*Research	0.85	0.76	0.80	0.55	0.53	0.42	0.61	0.65	0.60

RANK	RESEARCH QUESTION	Category	C1: Tech Poss	C2: Feas	C3: Issues of Use	C4: Scale	C5: Impact	C6: Equity	C7: Inn	RPS	AEA
126	Ask large crowds of civil society members and health workers to report violations of the International Code of Marketing of Breast Milk Substitutes both in society and in facilities.	Data generation	0.74	0.66	0.82	0.58	0.51	0.57	0.64	0.65	0.56
127	Use crowdsourcing to find out who has what data in a country around a specific issue.	Data generation	0.75	0.59	0.74	0.61	0.61	0.61	0.60	0.64	0.54
128	Use local-only crowds to set priorities in a CHNRI exercise (thereby avoiding the criticism that trans-national, foreign funders set the research agenda and make funds available but may be out of touch with what is actually necessary in a local context).	Problem-solving	0.76	0.72	0.90	0.58	0.60	0.41	0.54	0.64	0.57
129	Generate data on visits to medical centres to measure availabilities of services and where is best to send patients.	Data generation	0.92	0.86	0.85	0.43	0.48	0.45	0.51	0.64	0.62
130	Use large crowds of CHWs to report on substandard drugs distributed and use (e.g. antimalarials, TB drugs, bednets, RDTs and other medical commodities).	Data generation	0.78	0.54	0.74	0.62	0.69	0.60	0.52	0.64	0.57
131	Ask crowds of relevant experts/practitioners to generate ideas of how to deal with a number of humanitarian scenarios that are presented, where currently there are gaps in guidance and poor direction on 'how' to do programming.	Epidemic responses	0.61	0.84	0.86	0.60	0.56	0.48	0.54	0.64	0.55
132	Ask crowds of recipients of public health interventions to rate performance of CHWs and to report negligence and harm.	Problem-solving	0.78	0.80	0.82	0.56	0.43	0.47	0.63	0.64	0.56

RANK	RESEARCH QUESTION	Category	C1: Tech Poss	C2: Feas	C3: Issues of Use	C4: Scale	C5: Impact	C6: Equity	C7: Inn	RPS	AEA
133	Ask large crowds of local health workers to report by text message when they see a child with an illness, such as diarrhoea, malaria, acute malnutrition, or other such diseases, to gather real-time maps of areas that could be targeted for direction of resources and identification of epidemics.	Data generation	0.68	0.67	0.81	0.72	0.62	0.48	0.51	0.64	0.57
134	Create a new crowdfunded, crowdsourced grant system where population health experts identify problems needing research, potential research are identify to research these problems, crowdfunding is used to generate funds to fund these problems and the research approval process is crowdsourced using the same crowd in the first step.	*Research	0.80	0.77	0.75	0.55	0.48	0.51	0.63	0.64	0.58
135	Use large crowds of local health workers to identify needs for technical assistance and/or capacity development required for effective program implementation to generate a pool of information for further prioritization and program support.	Problem-solving	0.76	0.54	0.81	0.55	0.60	0.63	0.61	0.64	0.55
136	Ask clinicians and health experts for strategies for infection control in high-volume facilities with a focus on the labour ward.	Problem-solving	0.73	0.42	0.80	0.62	0.73	0.60	0.57	0.64	0.58
137	Use large crowds of health-related text messaging recipients to monitor impact on awareness and actual (rather than hypothetical) health practices.	Data generation	0.74	0.67	0.79	0.49	0.49	0.64	0.66	0.64	0.54
138	Have health workers SMS cold chain violations for vaccines and number of 'stock-outs' reported.	Data generation	0.69	0.80	0.76	0.65	0.58	0.49	0.50	0.64	0.59

RANK	RESEARCH QUESTION	Category	C1: Tech Poss	C2: Feas	C3: Issues of Use	C4: Scale	C5: Impact	C6: Equity	C7: Inn	RPS	AEA
139	Use two separate crowsourcing campaigns for morbidity and then a "capture-recapture" analysis to refine the actual prevalence.	Data generation	0.80	0.49	0.69	0.62	0.66	0.52	0.69	0.64	0.55
140	Use citizen reporting in an online system or through mobile text to a council/village database to develop vital registration systems where there are none.	Data generation	0.76	0.63	0.86	0.61	0.55	0.47	0.59	0.64	0.55
141	Develop a system where an information seeker (e.g. a patient of HCW in an LMIC) is automatically matched with a person somehwere on the planet who is likely to be able to answer their questions. Communication can be over video, audio or SMS, synchronous or asynchronous. Costs for data and 'expert' thime are covered through paying patients and/or donations. A good point system should be created to rewards users who consistently provide helpful answers to avoid having to pay answer-providers. If successful, such a system could enable rapid re-allocation of knowledge to wherever and whenever is needed. it could also be a way for medical students to gain experience.	Problem-solving	0.81	0.76	0.78	0.50	0.60	0.41	0.60	0.64	0.57
142	Use crowdsourcing to select data from medical institutions that is appropriate for sharing, remove mistakes and to make it available to be shared across medical institutions (and therefore more useful).	Problem-solving	0.87	0.80	0.94	0.55	0.40	0.41	0.48	0.64	0.61
143	Crowdsource pathology diagnosis to limit specialist's time. Specialists only need to confirm positive diagnosies (already found through crowdsourcing) rather than sort through findings.	Diagnostics	0.71	0.67	0.77	0.65	0.54	0.55	0.58	0.64	0.55

RANK	RESEARCH QUESTION	Category	C1: Tech Poss	C2: Feas	C3: Issues of Use	C4: Scale	C5: Impact	C6: Equity	C7: Inn	RPS	AEA
144	Ask large crowds of ordinary people in different parts of the world using popular social media about their top 5 most pressing health-related issues facing their communities today, in 10 years time and in 50 years time (e.g. water security, food safety, air pollution) and any ideas they have for overcoming them locally.	Problem-solving	0.65	0.48	0.87	0.65	0.58	0.65	0.57	0.64	0.54
145	Image-based diagnosis through crowd-sourcing images to distributed networks of experts, increasing the speed of diagnosis and removing the need for on-site experts, similar to crowdmed.com	Diagnostics	0.65	0.63	0.81	0.66	0.65	0.43	0.61	0.64	0.55
146	Carry out an SMS survey to explore what populations learn through serious gaming and general game playing to explore applications in healthcare topics, such as health education.	Problem-solving	0.68	0.64	0.77	0.52	0.51	0.61	0.70	0.63	0.58
147	To establish incentive competitions and prizes to crowdsource academia and the general public to use their creativity to solve tough healthcare challenges like high defaulter rates from treatment for conditions like severe acute malnutrition, focused antenatal care, routine immunization and also to reduce cost of drugs and therapeutic products like Ready-to-Use-Therapeutic Foods for treatment of acute malnutritions.	Problem-solving	0.88	0.48	0.90	0.54	0.43	0.54	0.64	0.63	0.60
148	Ask experts and service users (including HIV, TB, family planning, mental health, adolescent health services) what new ideas there are to effectively reduce the problem of stigma and discrimination that are undermining the uptake of many health services and how to sustain the change over time.	Problem-solving	0.79	0.63	0.80	0.53	0.44	0.60	0.61	0.63	0.56

RANK	RESEARCH QUESTION	Category	C1: Tech Poss	C2: Feas	C3: Issues of Use	C4: Scale	C5: Impact	C6: Equity	C7: Inn	RPS	AEA
149	Use big data on mobile phone calling patterns, location patterns and airtime purchasing patterns to determine economic status and its association with the health status of children under 5, thus providing an opportunity for targeted healthcare messages through SMS technology.	Data generation	0.65	0.48	0.72	0.63	0.66	0.70	0.56	0.63	0.54
150	Have patients suggest the outcomes from clinical trials. For example, outcomes in trials involving pregnancy and childbirth are often physiological and these outcomes are less important to the women studied.	Problem-solving	0.74	0.61	0.90	0.54	0.44	0.47	0.70	0.63	0.55
151	Ask crowds of recipients of public health interventions (i.e. pregnant mothers) to rate the quality of an interaction with the health care providers (e.g. CHWs, community health centres, etc.). The quality rated could range from reach, coverage, content and interaction.	Problem-solving	0.67	0.46	0.77	0.63	0.60	0.59	0.65	0.63	0.55
152	Use crowdsourcing to link up healthworkers from LMICs with the international community of health specialists in real-time to improve the quality of medical management of complex cases.	Problem-solving	0.85	0.68	0.81	0.54	0.49	0.39	0.61	0.63	0.56
153	Use large crowds of local public health managers involved in program implementation to report by SMS how they solve context-specific barriers or obstacles in real-time to create a database of potential solutions to program implementation.	Problem-solving	0.81	0.77	0.87	0.65	0.43	0.26	0.59	0.62	0.64
154	Asking health care providers in LMICs what the main patients safety threats are and how they can be solved.	Problem-solving	0.67	0.69	0.90	0.53	0.47	0.56	0.56	0.62	0.55

RANK	RESEARCH QUESTION	Category	C1: Tech Poss	C2: Feas	C3: Issues of Use	C4: Scale	C5: Impact	C6: Equity	C7: Inn	RPS	AEA
155	Use crowd sourcing to monitor quality of care stratified by type of provider and primary complaint (e.g. chronic cough, persistent vomiting or acute onset of drowsiness in under-five children, etc.).	Problem-solving	0.86	0.79	0.81	0.48	0.44	0.39	0.58	0.62	0.62
156	Have individuals volunteer a transaction 'gift' on mobile phone payments.	Problem-solving	0.72	0.64	0.81	0.58	0.53	0.50	0.59	0.62	0.55
157	Ask the crowd and mobile phones for public health messages (i.e. use the crowd to 'tell a friend' or 'tell a patient').	Problem-solving	0.80	0.74	0.85	0.41	0.48	0.44	0.63	0.62	0.57
158	Use large crowds of pharmaceutical indisutries and research institutions for generating ideas on addressing antibiotic/drug resistance of major communicable diseases (e.g. TB).	Problem-solving	0.78	0.71	0.77	0.61	0.49	0.44	0.55	0.62	0.56
159	Use large crowds of civil society members to provide periodic/regular information on local food prices (including healthy and unhealthy food products) and purchasing patterns to enable tracking of food availability, consumption and price trends.	Data generation	0.82	0.68	0.77	0.57	0.50	0.51	0.50	0.62	0.54
160	Ask large crowds of health workers for ways they can better deliver specific services; for example, how they can provide better services during antenatal checks and facility deliveries with adequate counselling time for mothers.	Problem-solving	0.62	0.64	0.77	0.63	0.42	0.62	0.64	0.62	0.56

RANK	RESEARCH QUESTION	Category	C1: Tech Poss	C2: Feas	C3: Issues of Use	C4: Scale	C5: Impact	C6: Equity	C7: Inn	RPS	AEA
161	Ask HCWs to provide real-time monitoring of drugs/stocks on key commodities via SMS by keeping an inventory of stock in kep programs (e.g. EPI 'how many vials of PCV do you have' - same for all key commodities.) Use this to support forecasting/procurement plans and commodity logistics, drug stock-out hot spots and to identify irregular use.	Data generation	0.63	0.49	0.83	0.68	0.64	0.54	0.52	0.62	0.54
162	Fund a one-year national effort in collaboration with national mass media to map environmental obstacles to public health. First, set up a reporitng system similar to Ushahidi that can receive geotagged reports. Then, each month during the year (or each week during a shorter time span), focus on reporting one core area such as food, water, sanitation, corruption or environmental hazards. Prior to project commencment, have a clear plan on analysis and follow-up and communicate this plan to public to incentivize reporting.	Data generation	0.71	0.62	0.84	0.44	0.40	0.71	0.60	0.62	0.58
163	Ask large crowds of community members to share information about access to services.	Data generation	0.83	0.69	0.86	0.47	0.45	0.43	0.58	0.62	0.56
164	Ask large crowds of mothers in order to better understand what they and their families/communities require in order to enable them to sustain positive health behaviour, for example, to breastfeed exclusively for 6 months.	Problem-solving	0.79	0.53	0.90	0.54	0.48	0.49	0.58	0.62	0.59

RANK	RESEARCH QUESTION	Category	C1: Tech Poss	C2: Feas	C3: Issues of Use	C4: Scale	C5: Impact	C6: Equity	C7: Inn	RPS	AEA
165	Illutrating cold chain challenges through real-time feedback from logisticis managers and staff (i.e. "this box doesn't fit in my fridge!") could help manufacturers package products better for real-world use.	Data generation	0.79	0.79	0.82	0.53	0.47	0.42	0.50	0.62	0.60
166	Use crowdsourcing to mobilise communities. For example 'show up Saturday morning to help dig a latrine' or 'show up Monday at the representative's office to protest the delayed delivery of drugs.'	Problem-solving	0.72	0.75	0.70	0.54	0.53	0.38	0.70	0.62	0.57
167	Ask clinicians and health experts for strategies to combat the overuse of antibiotics and antimicrobial resistance.	Problem-solving	0.79	0.56	0.75	0.54	0.53	0.50	0.64	0.62	0.56
168	Ask the crowd in places where there is no vital registration which potential systems would be most feasible.	Problem-solving	0.65	0.74	0.84	0.64	0.48	0.40	0.54	0.61	0.56
169	Ask crowds of ordinary people outside the health domain using social media and professional networks for ideas that could help reduce the world's human population and its environmental footprint locally, regionally and globally over a 5, 10, 20 and 50 year period.	Problem-solving	0.69	0.73	0.82	0.48	0.55	0.51	0.53	0.61	0.54
170	Ask health care providers what form of a specific intervention, for example, Rh immunoglobulin (IgG), is used in their communities. If health care providers are not using this intervention or cannot name the type of intervention used, the information from this combined with the estimated burden can then be used to estimate consequences to provide rationale for policy makers and funders to provide investments in these areas.	Data generation	0.71	0.69	0.68	0.54	0.48	0.55	0.65	0.61	0.56

RANK	RESEARCH QUESTION	Category	C1: Tech Poss	C2: Feas	C3: Issues of Use	C4: Scale	C5: Impact	C6: Equity	C7: Inn	RPS	AEA
171	Ask parents and caregivers in different regions when they think they would take their child to a health facility, what the challenges are with taking them and what the challenges are with following up post-discharge.	Problem-solving	0.64	0.44	0.80	0.53	0.61	0.68	0.60	0.61	0.52
172	Crowdsourcing for "health games," targeting to children that inform on health issues and give rewards such as mobile cell time.	Problem-solving	0.78	0.71	0.76	0.59	0.41	0.44	0.59	0.61	0.58
173	Run large epidemiological studies in areas that are typically underrepresented by having crowds volunteer to send in information about themselves.	Data generation	0.74	0.74	0.70	0.47	0.55	0.45	0.62	0.61	0.56
174	Use crowdsourcing to help determine the availability of basic facilities at health posts (such as running water, soap, toilets) which need to be monitored for the SDGs.	Data generation	0.75	0.62	0.67	0.53	0.61	0.44	0.64	0.61	0.54
175	Create a national or international mentorship program, through which inexperienced remotely located health workers can speak regularly to somebody with more experience on how to approach challenges they face in their local community.	Problem-solving	0.70	0.45	0.79	0.60	0.67	0.52	0.49	0.60	0.57
176	Ask mothers or caregivers of children who have recently had a disease/infection (e.g. diarrhoea) to send back an SMS on the problems encountered in the implementation of the programs at the community-levels (i.e. barriers to accessing care).	Data generation	0.86	0.71	0.86	0.56	0.38	0.34	0.52	0.60	0.63
177	Have local health workers involved in disaster and emergency response SMS needs to a central location equipped to respond.	Epidemic responses	0.90	0.79	0.69	0.62	0.42	0.32	0.46	0.60	0.60

RANK	RESEARCH QUESTION	Category	C1: Tech Poss	C2: Feas	C3: Issues of Use	C4: Scale	C5: Impact	C6: Equity	C7: Inn	RPS	AEA
178	Send mass emails to health staff to pool the top 3 problems or health services problems.	Problem-solving	0.70	0.61	0.69	0.71	0.59	0.37	0.54	0.60	0.55
179	Use large crowds to gauge response to mass media health message campaigns by having individuals report through SMS a health message they have seen or heard and whether they have followed through on an action or not.	Data generation	0.69	0.56	0.76	0.66	0.55	0.39	0.58	0.60	0.51
180	Use crowdsourcing to trace the complex funding of global health.	Problem-solving	0.78	0.76	0.65	0.53	0.53	0.35	0.59	0.60	0.55
181	Despite the wealth of high quality research published in peer-reviewed journals on strengthening health systems, little of this research has translated into policy and every day practice in an LMIC-context. Ask those working in an LMIC-contect how to better integrate high-quality research into the primary healthcare systems into their context.	Problem-solving	0.81	0.65	0.82	0.54	0.45	0.37	0.56	0.60	0.54
182	Crowdsource potential drug targets for underserved diseases by making a game (like Foldit for protein folding) that allows users to try to match molecules to active sites.	Novel discoveries	0.76	0.62	0.78	0.47	0.45	0.56	0.54	0.60	0.52
183	Ask caregivers or mothers what food their child eats and the average daily food consumption patterns in order to establish eating behavious in children. This will provide valuable information that can be used to tackle undernutrition and obesity.	Data generation	0.71	0.71	0.66	0.60	0.45	0.49	0.57	0.60	0.54
184	Create an anonymous reporting system for people who have paid bribes and how much they paid to counter corruption. Use the data to create a heatmap. Authorities can follow-up on the reports	Data generation	0.68	0.56	0.76	0.60	0.50	0.43	0.65	0.60	0.54

RANK	RESEARCH QUESTION	Category	C1: Tech Poss	C2: Feas	C3: Issues of Use	C4: Scale	C5: Impact	C6: Equity	C7: Inn	RPS	AEA
	that have been taken.										
185	Develop a smartphone app targeted at women and girls that includes educational materials but also enables women to ask for help in a secure, non-traceable and anonymous way. The app should be usable for women and girls with only basic levels of literacy.	Problem-solving	0.53	0.76	0.86	0.55	0.54	0.44	0.47	0.60	0.52
186	Use crowdsourcing for ideas, like giant brainstorming. For example, "Citizens of Baltimore, please provides ideas about how we can constructively work with policy to bring calm to our city."	Problem-solving	0.59	0.77	0.73	0.50	0.50	0.45	0.60	0.59	0.49
187	Ask people what are the different components of reproductive health (as a means to see if they are aware that it is more than just pregnancy and childbearing).	Problem-solving	0.68	0.53	0.88	0.50	0.47	0.46	0.58	0.59	0.52
188	Ask large crowds of health providers (at a national level) and program managers (in multiple countries) to report on availability/provision of new interventions introduced in a country/province/region (e.g. WHO IMCI Guidelines have not been introduced or scaled up although introduced in 2014).	Problem-solving	0.78	0.59	0.80	0.48	0.49	0.47	0.49	0.59	0.55
189	Submit reports via text message based on a check-list of support supervision, including stock out of tracer drugs and reasons, in countries whre supportive supervision is standardized through use of integrated checklists.	Data generation	0.72	0.77	0.79	0.37	0.41	0.55	0.49	0.59	0.57

RANK	RESEARCH QUESTION	Category	C1: Tech Poss	C2: Feas	C3: Issues of Use	C4: Scale	C5: Impact	C6: Equity	C7: Inn	RPS	AEA
190	Have mothers communicate their children's top 3 health needs to local/national health officials (via SMS, Twitter or other social media).	Data generation	0.61	0.45	0.88	0.50	0.53	0.56	0.53	0.58	0.52
191	Teach laypersons to recognize common skin findings by looking at pictures, then crowdsource diagnostics of these skin conditions to the crowd.	Diagnostics	0.91	0.60	0.79	0.49	0.43	0.39	0.46	0.58	0.59
192	Ask the HCWs the challenges they face and then use responses to guide the development of apps to solve challenges identified (e.g. apps for disease diagnosis and mangement).	Data generation	0.59	0.59	0.82	0.61	0.45	0.46	0.54	0.58	0.51
193	Ask both clinicians and women how to prevent the overuse of unnecessary caesarean sections.	Problem-solving	0.58	0.62	0.68	0.62	0.59	0.40	0.56	0.58	0.52
194	Create partnerships with media houses and health care providers involving social marketing to target child health promotion in poor rural communities through the utilization of blogs and apps as reminders for appointments for maternal, child and infant health check-ups.	Problem-solving	0.79	0.43	0.93	0.49	0.44	0.37	0.61	0.58	0.59
195	Use a large crowd of health workers and combine the verbal autopsy methodology and mHealth technology to explore what they think 'went wrong' on specific problems, such as in the context of HIV care and other health-related issues.	Data generation	0.71	0.62	0.83	0.48	0.33	0.47	0.60	0.58	0.59
196	Use crowdsourcing as part of programme monitoring and evaluation to give qualitative detail to help interpret other evaluation data.	Data generation	0.76	0.68	0.79	0.44	0.38	0.40	0.58	0.58	0.56
197	Ask health care providers, patients and caregivers in LMICs about strategies to improve dementia care.	Problem-solving	0.67	0.68	0.85	0.49	0.47	0.23	0.64	0.57	0.55

RANK	RESEARCH QUESTION	Category	C1: Tech Poss	C2: Feas	C3: Issues of Use	C4: Scale	C5: Impact	C6: Equity	C7: Inn	RPS	AEA
198	Use crowdsourcing to identify and link pregnant women in the community to CHWs without compromising privacy.	Problem-solving	0.68	0.42	0.71	0.47	0.68	0.52	0.53	0.57	0.55
199	Use crowdsourcing for global mapping of (infectious) diseases, including enhanced pharmaco-surveillance by any individual starting a medical treatment.	Data generation	0.69	0.60	0.84	0.44	0.52	0.41	0.50	0.57	0.53
200	Crowdsource information in order to better understand behaviour change, including how to change behaviour and what the most effective communication channels and methods that lead to behaviour change (e.g. use citizen self reporting or health working data to crowdsource information and map areas with high vaccination coverage and come up with solutions for vaccine coverage in real-time).	Problem-solving	0.82	0.55	0.76	0.48	0.37	0.40	0.61	0.57	0.55
201	Have a paper-based 'suggestion box' in a health facility that is regularly pick up and USED. This is more anonymous than cell phone technology and more accessible, since the Internet is not universally accessible.	Data generation	0.73	0.57	0.75	0.45	0.52	0.39	0.58	0.57	0.54
202	Ask the crowd to grade large numbers of diagnostic medical images obtained in the community which would represent a significant reduction in the need for specialist grades (i.e. a means to develop daibetic retinopathy grading programs in LMICs)	Diagnostics	0.85	0.76	0.78	0.41	0.40	0.25	0.53	0.57	0.62

RANK	RESEARCH QUESTION	Category	C1: Tech Poss	C2: Feas	C3: Issues of Use	C4: Scale	C5: Impact	C6: Equity	C7: Inn	RPS	AEA
203	Crowdsourced data set collection. Medical experts are asked to record and submit symptoms, timelines, disease progression, medications prescribed, etc. Over time, a vast repository is collected, containing the necessary statistics and prior data on diseases. This can be used to automate and significantly improve diagnosis, detect new diseases and improve treatment.	Data generation	0.61	0.56	0.77	0.42	0.52	0.51	0.58	0.57	0.51
204	Use crowdsourcing to ascertain changes in gender ratios over time amongst health staff (e.g. how many female doctors).	Data generation	0.68	0.58	0.68	0.59	0.46	0.50	0.48	0.57	0.49
205	Crowdsource analysis of satellite images after disasters to map extent of damage.	Epidemic responses	0.64	0.43	0.82	0.61	0.52	0.41	0.52	0.56	0.52
206	Use the Internet (Amazon Mechanical Turk and other resourcecs) to survey large populations in remote areas about disease/symptom prevalence, access to resources/care, living conditions, etc.	Data generation	0.81	0.52	0.74	0.45	0.39	0.51	0.52	0.56	0.56
207	Survey health care workers for their opinions on the utility of specific treatments, for example, whether there is a need to supplement zinc in the treatment of pneumonia.	Data generation	0.69	0.62	0.71	0.54	0.41	0.42	0.55	0.56	0.53
208	Ask the crowd what else can be delegated to CHWs in the field after competency-based training separately for each field (maternal and child health, family planning, communicable diseases, etc.)	Problem-solving	0.55	0.59	0.86	0.46	0.48	0.45	0.54	0.56	0.48

RANK	RESEARCH QUESTION	Category	C1: Tech Poss	C2: Feas	C3: Issues of Use	C4: Scale	C5: Impact	C6: Equity	C7: Inn	RPS	AEA
209	Create a WHO-operated database to which national centres can report their ongoing investigations, their results after completion and a contact person. The incentive for the individual research centre to report their studies is that they are alerted whenever others initiate or conclude research related to their own investigations. The greated difficulty would likely be to find a simple standard for a reporting format that would capture the goals of ongoing research.	*Research	0.63	0.65	0.75	0.60	0.46	0.42	0.39	0.56	0.53
210	Use crowdsourcing to link up health workers from LMICs with the international community of health workers to improve the professional training of the former.	Education	0.63	0.68	0.55	0.48	0.62	0.35	0.59	0.56	0.51
211	Ask large crowds of the general population what their source of drinking water is and whether they treat it before drinking. This will provide a pool of valuable information on behaviour and help formulate strategies to improve these practices in LMICs.	Data generation	0.52	0.60	0.73	0.55	0.65	0.34	0.49	0.55	0.53
212	Crowdsourcing to report actions, public statements and behaviour of politicians that are relevant to current health politicies.	Data generation	0.71	0.56	0.86	0.52	0.36	0.36	0.49	0.55	0.57
213	Ask providers in areas not covered by vital registration or routine surveillance, such as the HMIS system, to identify the top five causes of death across age groups in their area and use geospatial mapping to map differences in deaths by area.	Data generation	0.72	0.48	0.74	0.48	0.50	0.35	0.60	0.55	0.51

RANK	RESEARCH QUESTION	Category	C1: Tech Poss	C2: Feas	C3: Issues of Use	C4: Scale	C5: Impact	C6: Equity	C7: Inn	RPS	AEA
214	Use crowdsourcing to map capacity against need. For example, see if staff who have been trained in CLTS are stationed in areas where there is higher prevalanece of open defecation or staff who have been trained in epidemic preparedness are the ones handling an epidemic outbreak.	Problem-solving	0.72	0.80	0.60	0.45	0.36	0.39	0.47	0.54	0.55
215	Have local health workers delivering immunization services in a campaign SMS needs (e.g. supplies, refusals, etc.) to campaign leaders as they occur in real-time.	Problem-solving	0.60	0.54	0.77	0.59	0.43	0.31	0.55	0.54	0.53
216	Use crowds to prevent gender-based violence through crime mapping.	Data generation	0.62	0.68	0.69	0.61	0.50	0.21	0.47	0.54	0.52
217	Ask society about the benefits of a specific health field, condition or treatment (i.e. family planning, ORS, zinc) as a way to guage levels of awareness of the health benefits.	Data generation	0.68	0.54	0.78	0.49	0.39	0.35	0.55	0.54	0.52
218	Use SMS, email and social media messages to invite suggestions and identify management challenges from field level program managers on implementation challenges to identify questions for implementation science and operations research.	Problem-solving	0.64	0.64	0.69	0.39	0.44	0.45	0.52	0.54	0.49
219	Use large crowds of local health workers to collect information through SMS on why local health workers do not prescribe or do not believe in evidence-based interventions. Find what information they need in order to believe in it.	Problem-solving	0.83	0.65	0.70	0.42	0.35	0.29	0.50	0.53	0.57
220	Gather ideas on global solutions to global health problems through videos that can be rated (on Youtube?).	Problem-solving	0.71	0.71	0.83	0.40	0.30	0.32	0.46	0.53	0.57

RANK	RESEARCH QUESTION	Category	C1: Tech Poss	C2: Feas	C3: Issues of Use	C4: Scale	C5: Impact	C6: Equity	C7: Inn	RPS	AEA
221	Use large crowds of non-medical specialists connected to the internet to solve simple micro-tasks related to the analysis of medical data, such as identifying particular pathologic patterns in microscopy images.	Problem-solving	0.64	0.51	0.65	0.50	0.44	0.37	0.57	0.53	0.48
222	Request program managers from several countries or regions to provide their quality improvement approaches, use these to narrow down which approaches to look at in detail.	Problem-solving	0.81	0.62	0.77	0.36	0.31	0.26	0.52	0.52	0.61
223	Crowdsource data about morbidities by individuals with given conditions. For example, map diabetes or upload serial blood sugar information.	Data generation	0.45	0.56	0.71	0.59	0.53	0.42	0.36	0.52	0.51
224	Have a competition through Coca Cola, or a similar company, where individuals are asked in any country to give three local ways of making nutritious food cheaply in their area.	Problem-solving	0.50	0.46	0.75	0.43	0.43	0.51	0.54	0.52	0.48
225	Use crowdsourcing to revamp vital registration in areas that do not have VR systems in place and also by cross-checking information for quality assurance in areas that have deficient VR systems. For example, individuals (health workers and members of the community -- informed via media campaigns) would be encouraged to report births and deaths by text message and an automated server would take care of gathering necessary information to characterize the event (by using a decision tree that would reply with the relevant follow-up questions) and to reconcile duplicated reports of events and also verifying accuracy and veracity by texting other phones in the	Data generation	0.64	0.32	0.68	0.59	0.54	0.50	0.35	0.52	0.51

RANK	RESEARCH QUESTION	Category	C1: Tech Poss	C2: Feas	C3: Issues of Use	C4: Scale	C5: Impact	C6: Equity	C7: Inn	RPS	AEA
	area (identified via geolocation) to verify the information.										
226	Use crowdsourcing to monitor trends in scale-up and coverage of newly introduced vaccines and micronutrients.	Data generation	0.45	0.59	0.65	0.59	0.52	0.37	0.40	0.51	0.48
227	Ask large crowds of local health workers (or whatever cadre of workers provides vaccinations) to report by SMS the children that they vaccinate each day to generate real-time data for vaccine coverage.	Data generation	0.46	0.62	0.77	0.45	0.36	0.45	0.46	0.51	0.55
228	Monitor phase IV drug trials through crowdsourcing.	Research	0.58	0.58	0.59	0.41	0.52	0.29	0.61	0.51	0.49
229	Ask large crowds of health workers to better understand why young children have poor appetites and do not grow well.	Data generation	0.62	0.41	0.56	0.52	0.42	0.52	0.50	0.51	0.50
230	Use crowdsourcing to help annotate medical data, find errors, outliers, etc., and use this to train algorithms.	Data generation	0.50	0.70	0.52	0.54	0.48	0.39	0.39	0.50	0.50

RANK	RESEARCH QUESTION	Category	C1: Tech Poss	C2: Feas	C3: Issues of Use	C4: Scale	C5: Impact	C6: Equity	C7: Inn	RPS	AEA
231	Use large crowds of local health experts to collect through SMS what prevents vulnerable women and chlidren from using basic health services, such as breastfeeding, ORS and immunization. Use this information to develop approaches to increase awareness, acceptance and utilization of these services.	Problem-solving	0.61	0.65	0.66	0.44	0.40	0.34	0.38	0.50	0.54
232	Ask the general public to submit short code SMS to report malpractice in the health sector, such as illegal under the counter fees, bribes, health staff absenteeism, inappropriate behaviour, etc. (similar to U-report in Uganda).	Data generation	0.64	0.42	0.71	0.56	0.42	0.14	0.54	0.49	0.57
233	Create an organisation like Avon where women come to the home to do monthly product descriptions (like 'kitchen parties') and ask women in those groups to say what they think would make the biggest difference to their children's health.	Problem-solving	0.62	0.48	0.62	0.46	0.36	0.36	0.50	0.49	0.49
234	Use large crowds of community leaders to report vaccine preventable diseases by SMS.	Data generation	0.61	0.43	0.57	0.54	0.52	0.23	0.51	0.49	0.53
235	Ask large crowds of global health experts to provide materials (reagents, equipment, etc.) they no longer use or need but that could be redistributed in places where they are needed.	Problem-solving	0.65	0.68	0.57	0.40	0.39	0.24	0.43	0.48	0.57
236	HCWs could text photos of suspected cases of NTDs to specialists, yielding population-level data on incidence. Families can be asked to provide dietary information; this can be used to identify additional foods to be fortified.	Data generation	0.74	0.49	0.77	0.39	0.29	0.26	0.39	0.47	0.58

RANK	RESEARCH QUESTION	Category	C1: Tech Poss	C2: Feas	C3: Issues of Use	C4: Scale	C5: Impact	C6: Equity	C7: Inn	RPS	AEA
237	Give mothers around the world extra phone top-up credit for submitting good and plausible ideas of how to improve their children's health.	Problem-solving	0.64	0.44	0.51	0.45	0.31	0.35	0.48	0.45	0.49
238	Use crowdsourcing to locate unpublished research and evaluation results and ongoing research (who's doing what before it's published).	*Research	0.51	0.41	0.59	0.43	0.40	0.30	0.44	0.44	0.55
239	Ask patients and families where they are seeking care, why and how much they are spending.	Data generation	0.60	0.41	0.69	0.26	0.38	0.28	0.31	0.42	0.58
